# Small Rho GTPases and the Effector VipA Mediate the Invasion of Epithelial Cells by Filamentous *Legionella pneumophila*

**DOI:** 10.3389/fcimb.2018.00133

**Published:** 2018-05-03

**Authors:** Akriti Prashar, María Eugenia Ortiz, Stefanie Lucarelli, Elizabeth Barker, Zohreh Tabatabeiyazdi, Feras Shamoun, Deepa Raju, Costin Antonescu, Cyril Guyard, Mauricio R. Terebiznik

**Affiliations:** ^1^Department of Biological Sciences, University of Toronto at Scarborough, Scarborough, ON, Canada; ^2^Department of Cell and Systems Biology, University of Toronto, Toronto, ON, Canada; ^3^Department of Chemistry and Biology, Ryerson University, Toronto, ON, Canada; ^4^Bioaster, Lyon, France; ^5^Molecular Microbiology, Public Health Ontario, Toronto, ON, Canada

**Keywords:** *Legionella pneumophila*, Rho GTPases, actin, VipA, filamentous bacteria

## Abstract

*Legionella pneumophila* (Lp) exhibits different morphologies with varying degrees of virulence. Despite their detection in environmental sources of outbreaks and in respiratory tract secretions and lung autopsies from patients, the filamentous morphotype of Lp remains poorly studied. We previously demonstrated that filamentous Lp invades lung epithelial cells (LECs) and replicates intracellularly in a *Legionella* containing vacuole. Filamentous Lp activates β1integrin and E-cadherin receptors at the surface of LECs leading to the formation of actin-rich cell membrane structures we termed hooks and membrane wraps. These structures entrap segments of an Lp filament on host cell surface and mediate bacterial internalization. Here we investigated the molecular mechanisms responsible for the actin rearrangements needed for the formation and elongation of these membrane wraps and bacterial internalization. We combined genetic and pharmacological approaches to assess the contribution of signaling downstream of β1integrin and E-cadherin receptors, and Lp Dot/Icm secretion system- translocated effectors toward the invasion process. Our studies demonstrate a multi-stage mechanism of LEC invasion by filamentous Lp. Bacterial attachment to host cells depends on signaling downstream of β1integrin and E-cadherin activation, leading to Rho GTPases-dependent activation of cellular actin nucleating proteins, Arp2/3 and mDia. This mediates the formation of primordial membrane wraps that entrap the filamentous bacteria on the cell surface. Following this, in a second phase of the invasion process the Dot/Icm translocated effector VipA mediates rapid membrane wrap elongation, leading to the engulfment of the filamentous bacteria by the LECs. Our findings provide the first description of Rho GTPases and a Dot/Icm effector VipA regulating the actin dynamics needed for the invasion of epithelial cells by Lp.

## Introduction

*Legionella pneumophila* (Lp), the etiological agent of Legionnaires' disease, is an intracellular pathogen found ubiquitously in natural and man-made aquatic systems, where it thrives inside protozoa and forms biofilms (McDade et al., 1977; Fields, 1996; Steinert et al., 2002). A majority of studies examining Lp pathogenicity have focused on the invasion and intracellular replication of the bacteria in macrophages. These studies have identified the role of several Dot/Icm type IV secretion system (T4SS) translocated effectors that modify the bacteria-containing phagosome into a replication permissive compartment known as the *Legionella* containing vacuole (LCV) (Ensminger, 2015). Along with macrophages, alveolar epithelial cells may also play an important role in Legionnaires' disease. Indeed, the ability of Lp to infect lung epithelial cells (LECs) has been described using different models of infection, including human lung explants (Daisy et al., 1981; Mody et al., 1993; Cianciotto et al., 1995; Newton et al., 2010; Brown et al., 2013; Jäger et al., 2014).

Lp has a complex life cycle in which it develops different morphologies with varying capacities for extracellular survival and intracellular replication (Garduno et al., 2008; Robertson et al., 2014). Among Lp morphotypes, the filamentous form remains poorly studied, in spite of being found in cultured mammalian cells (Ogawa et al., 2001; Garduño et al., 2011; Prashar et al., 2012, 2013), biofilms (Piao et al., 2006) and sputum, bronchoalevolar lavage and histological specimens from patients with legionnaires' disease (Blackmon et al., 1978; Boyd et al., 1978; Rodgers, 1979; Hernandez et al., 1980; Legionella Molecular Biology, 2008; Prashar et al., 2012). We have previously shown that filamentous Lp can invade LECs and macrophages and these intracellular filaments undergo fragmentation to produce bacillary infectious progeny (Prashar et al., 2012, 2013).

The invasion of LECs by filamentous Lp occurs via a process that resembles a case of the zipper mechanism of invasion known as overlapping phagocytosis (Rittig et al., 1998, 1999; Prashar et al., 2012), which has been described for the uptake of *Francisella tularensis* and *Candida albican*s (d'Ostiani et al., 2000). The invasion is initiated by the binding and activation of host cell β1integrin and E-cadherin receptors at the cell surface by unknown bacterial adhesins (Prashar et al., 2012). However, unlike other bacteria, where the binding of adhesins to receptors is sufficient to anchor the pathogens to the host cells (Young et al., 1992; Tang et al., 1994; Cowan et al., 2000; Sa et al., 2010), the attachment of filamentous Lp to LECs is a more complex process, which is likely a consequence of the bacterial morphology. The attachment requires the formation of filopodial and lamellar structures, which we named “hooks” and “membrane wraps,” respectively, that entrap segments of the filaments on the host cell surface (Prashar et al., 2012). These membrane wraps elongate over time through a process that depends on actin polymerization, encompassing longer segments of the filament in a “pre-vacuolar” compartment. The entrapped segments of Lp filaments subsequently lose their association with F-actin and the bacteria are internalized in an LCV, where they fragment to produce short rods that replicate (Prashar et al., 2012). The cellular and molecular mechanisms driving actin remodeling during filamentous Lp attachment and entry in LECs are not fully understood.

The subversion of the host cell actin cytoskeleton is a common strategy used by bacterial pathogens to force their uptake by non-phagocytic cells (Knodler et al., 2001; Zhou and Galan, 2001; Rottner et al., 2005; Schlumberger and Hardt, 2005; Sousa et al., 2007; Huveneers and Danen, 2009; Ireton et al., 2014; Bugalhão et al., 2015). Src tyrosine kinase (Src), class I phosphoinositide 3-kinase (PI3K) and Rho family of small GTPases (hereafter referred to as Rho GTPases) are among the host targets exploited by pathogenic bacteria to achieve this objective (Finlay, 2005; Heasman and Ridley, 2008; Hall, 2012). In this study, we show that the attachment and internalization of filamentous Lp in LECs result from the combined action of both, β1integrin and E-cadherin downstream signaling cascades and the T4SS effector VipA, an actin nucleating protein (Franco et al., 2012). The engagement of β1integrin and E-cadherin receptors by filamentous Lp leads to Src activation, PI3K signaling and the activation of the Rho GTPases, Cdc42, Rac1 and RhoA, that in turn induce the formation of membrane wraps for the effective entrapment of filamentous Lp. The T4SS effector VipA is then translocated into the host cells, favoring the elongation of membrane wraps leading to a faster internalization of bacterial filaments by the epithelial cells.

Our results show, for the first time, that host Rho GTPase signaling is required for the invasion of LECs by filamentous Lp and this host cell signaling, together with the T4SS effector VipA mediates the invasion process.

## Materials and methods

### Reagents and constructs

Anti-*Legionella* antibody was from Public Health Ontario and anti-VipA antibody was generously provided by Dr. H Shuman (University of Chicago, USA). pSrc (Y416), total Src, total Akt antibodies were from Cell Signaling (Danvers, MA, USA) and the pAkt (S743) antibody was from ThermoFisher (Life technologies, Carlsbad, CA, USA). Anti-calnexin antibody was from BD biosciences (Mississauga, ON, Canada). FuGENE (HD) was from Promega Biosciences (Madison, WI, USA).

The following inhibitors were used in this study: PP2 (25 μM, Tocris) (Hanke et al., 1996), Ly294002 (100 μM, Sigma) (Vlahos et al., 1994), membrane permeable C3 transferase (0.5 μg/mL, Cytoskeleton Inc.) (Ridley and Hall, 1992), ML141 (20 μM, Tocris) (Surviladze et al., 2010), Blebbistatin (200 μM, Sigma) (Straight et al., 2003), Nsc23766 (50 μM, Tocris) (Gao et al., 2004), ROCK (1 μM, Millipore) (Narumiya et al., 2000), SMIFH2 (25 μM, Millipore) (Rizvi et al., 2009), CK-666 (80 μM, Sigma) (Nolen et al., 2009).

### Plasmids and oligonucleotides

Rac1-GFP, RhoA-GFP, PAK-PBD GFP and rGBD-GFP were kind gifts from Dr. Sergio Grinstein (The Hospital for Sick Children, Toronto, Canada) and Cdc42-GFP was from Dr. Katalin Szaszi (St. Michael's Hospital, Toronto, Canada). PH-Akt-GFP was a gift from Dr. Roberto Botelho (Ryerson University, Toronto, Canada) and has been previously described in Kontos et al. (1998). FH1/FH2-GFP were kind gifts from Dr. Andras Kapus (St. Michael's Hospital, Toronto). Plasmids and DNA fragment used to generate *vipA* mutant and complementation strains were generous gifts from Dr. H Shuman (University of Chicago, USA) and were previously described in Franco et al. (2012).

Primers for sequencing to confirm *vipA* deletion:

F- 5′ to 3′: GAACGCGCTTCAGTATGACA

R- 5′ to 3′: AGCATTGGCCTTTTGAGATA.

### Bacterial strains and culturing

*Legionella pneumophila* strain Lp02 was used in this study. *dotA* mutant strain was originally obtained from Dr. R Isberg (Tufts University Medical School, USA) (Berger and Isberg, 1993). RFP-Lp02 and RFP-*dotA* strains have been described previously in Prashar et al. (2012, 2013). *vipA* deletion mutant was generated in Lp02 strain as described in Franco et al. (2012). Briefly, bacteria were naturally transformed with a DNA fragment containing PCR products of kanamycin cassette and *vipA* flanking regions. A strain with IPTG-inducible expression of wild-type or mutant *vipA* allele were generated by natural transformation of *vipA* deletion mutants as described in Franco et al. (2012). Plasmids pMMB207c-*Ptac-vipA*^+^and Δ*vipA* pMMB207c-*Ptac-vipA-1* for these strains were generously provided by Dr. H Shuman (University of Chicago, USA) and were previously described in Franco et al. (2012). All strains were cultured as described previously in Prashar et al. (2012). Bacteria from frozen glycerol stocks were streaked on buffered charcoal yeast extract (BCYE) plates and allowed to grow for 3–4 days at 37°C at 5% CO_2_. Colonies were resuspended in buffered yeast extract (BYE) media and cultured for 24 h at 100 rpm at 37°C, followed by sub-culturing for 16–18 h till cultures reached OD_600_ of 3.5–4.0 (Molofsky et al., 2005). All experiments were performed with these late-exponential phase cultures. IPTG (1 mM) was added to the broth cultures to induce vipA or vipA-1 expression in assays using *vipA* complementation studies.

### Cell culturing and transfections

Unless otherwise stated, all experiments were performed using human alveolar epithelial cells NCI-H292 (ATCC CRL-1848). Cells were maintained in RPMI-1640 supplemented with 10% fetal bovine serum (FBS) (Wisent, Quebec) at 37°C at 5% CO_2_. Cells were cultured and transfected with indicated constructs using FugeneHD for at least 18 h before being used in experiments. MDCK cells (Madin-Darby Canine Kidney) (ATCC CCL-34) were maintained in Dulbecco's modified Eagle's media (DMEM) media supplemented with 10% FBS (Wisent, Quebec) at 37°C at 5% CO_2_. 16HBEo14o- cells were originally from Dr. Dieter C. Gruenert (Cardiovascular Research Institute, University of California, San Francisco, USA) (Gruenert et al., 1995). Cells were grown in Eagle's Minimum Essential Media (EMEM) supplemented with 10% FBS at 37°C at 5% CO_2_.

### Attachment assays

All infections were performed using post-exponential phase cultures as described above (in bacterial strains and culturing). 1.0 × 10^5^ cells were plated for 48 h, to yield approximately 2.0 × 10^5^ cells per well and infected with 6 × 10^7^ bacteria for 1 h at 37°C at 5% CO_2._ Unbound bacteria were washed 3 times with 1X PBS and cells were fixed. Under these conditions, 24.9 ± 2.7 bacteria attached to 100 cells. Where indicated, cells were treated with pharmacological inhibitors for specified times prior to the addition of bacteria. The inhibitors were maintained during the duration of the attachment assays.

### Internalization assays

Bacteria were allowed to attach for 1 h and unbound bacteria were washed. Internalization of attached bacteria was allowed to proceed for indicated times before cells were washed and fixed. External bacteria were immunolabeled using anti-Lp antibodies followed by permeabilization of cells with 0.1% Triton-X in 1XPBS. Actin was stained with Alexa fluorophore-conjugated phalloidin to label the membrane wraps. Internalization was quantified by measuring the lengths of the bacteria that were entrapped in the membrane wraps and were inaccessible to externally applied antibodies. Each segment of an Lp filament undergoing internalization was considered as a distinct internalization event instead of adding the lengths together to ensure that effects of treatments on membrane wrap elongation were not overlooked. For studies examining Lp internalization in the presence of pharmacological inhibitors, bacteria were allowed to attach to cells for 1 h. Unbound bacteria were washed and the media was replaced with media containing the indicated inhibitors for the duration of the experiment, followed by washing and fixation. Internalization was assessed by described above.

### Disruption of tight junctions

Low calcium treatments were performed as described previously in Pentecost et al. (2006). Confluent, polarized monolayers of MDCK cells were maintained in low calcium media (140 mM NaCl, 20 mM HEPES, pH 7.4, 3 mM KCl, 1 mM MgCl_2_, 5 mM glucose and 5 μM CaCl_2_) for 1 h at 37°C at 5% CO_2_. Cells were then switched back to normal DMEM media with 1.8 mM calcium and infected with Lp at MOI 300 for 10 min, washed and fixed. Attachment was quantified by manually enumerating the number of bacteria attached to cells.

For cell wounding assays, confluent MDCK monolayers were wounded using a 21 mm gauge needle and washed twice with PBS before being infected with RFP-Lp for 10 min, washed and fixed. Automated analysis was performed to quantify bacterial attachment to confocal micrographs of fields of cells using Volocity (PerkinElmer). With the freehand drawing function, images were cropped such that only areas close to the wound were included. Automatic threshold function was used to threshold the fluorescence signal for the bacteria between images. Mean intensity of channel corresponding to the RFP channel was measured to assess the number of bacteria attached to each field.

### Cytochalasin-D treatment for detachment assay

Cells were infected with the indicated strains for 1 h, unbound bacteria washed and infection allowed to proceed. 6 h p.i cells were washed and treated with DMSO or 10 μM cytochalasin-D for 20 min. Cells were washed with 1X PBS, fixed with 4% PFA and external bacteria were immunolabeled using anti-Lp antibodies.

### CFU enumeration

LECs were infected at MOI 300 as described above for 1 h. Unbound bacteria were washed and infection was allowed to proceed for an additional 17 h. All Lp strains used were auxotrophic for thymidine, therefore, 50 μg/ml of thymidine was maintained during the duration of the assay. IPTG (0.2 mM) was used in addition to thymidine to induce the expression of vipA in *vipA*-1 complemented strain. 18 h p.i cells were washed 3 times with 1X PBS and incubated with RPMI-1640 + 10% FBS + 100 μg/ml gentamicin for 1 h at 37°C/5% CO_2_. Cells were washed 3 times with 1X PBS followed by 15 min incubation with 0.1% saponin at 37°C at 5% CO_2_. Lysed cells were retrieved and serial dilutions were plated on BCYE + thymidine plates and viable bacteria were counted 3 days later.

### Immunofluorescence and microscopy

Confocal images were acquired using a spinning disc confocal microscope (Quorum Technologies) consisting of an inverted fluorescence microscope (DMI6000B; Leica) equipped with an ORCA-R2 camera and spinning disc confocal scan head, an ASI motorized XY stage and a Piezo Focus Drive (Quorum Technologies). Images were acquired using MetaMorph software (Molecular Devices). Data processing and analysis was performed using Volocity software (PerkinElmer) and images were prepared using Adobe Photoshop and Illustrator (Adobe Systems, Inc.). Where indicated images acquired as described above were deconvolved using calculated point spread function in Volocity. Unless stated otherwise, for all fluorescence images shown, the main panels are merged z-stacks and the associated higher magnifications are single confocal planes.

### Whole cell lysates and immunoblotting

At indicated times p.i, whole cell lysates were prepared using 1X Laemmli Sample Buffer (0.5 M Tris pH 6.8, Glycerol, 10% SDS, 10% β-mercaptoethanol, and 5% bromophenol blue). Proteins were resolved by Glycine-Tris SDS-PAGE followed by transfer onto a PVDF membrane, which were washed, blocked and incubated with antibodies as previously described (Garay et al., 2015). The levels of phosphorylated proteins were determined using ImageJ, following normalization to total protein expression (i.e. pSrc/total Src and pAkt/total Akt).

### Quantification and statistical analysis

Unless stated otherwise, all data shown are mean ± SEM from three independent experiments. Bacterial attachment to cells following inhibitor treatments was normalized to vehicle treated cells, for each replicate, expressed as 100%. Quantitative analysis of western blots was performed using ImageJ (NIH). All other quantifications were performed using fluorescence microscopy. Statistical analysis was performed using two-tailed Student's *t-test* when comparing means between 2 groups. Means between 3 or more groups were compared using one-way ANOVA with Tukey's multiple comparison tests (Graph Pad Prism software). Ninety-five percentage confidence interval was used to determine statistical significance.

## Results

### Src and PI3 kinase signaling is needed for attachment of filamentous Lp to LECs

**Figure 1:**
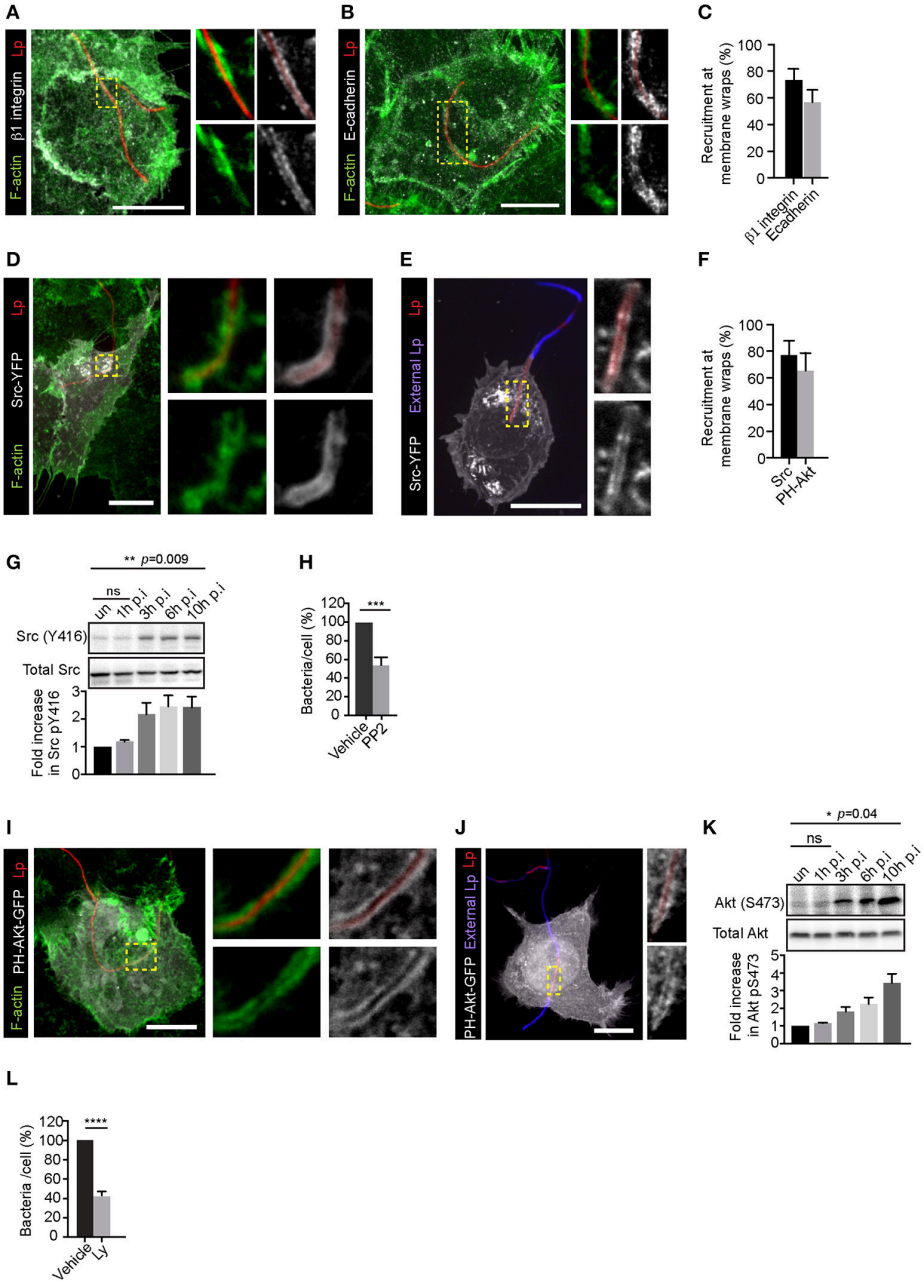
Attachment of filamentous Lp to LECs requires Src and PI3K activity. Representative confocal micrographs showing the recruitment of β1integrin **(A)** and E-cadherin **(B)** receptors at the membrane wraps 2 h post infection (p.i.). Green: F-actin, Grayscale: receptors, Red: Lp. Panels to the right show higher magnifications of framed regions. **(C)** Quantification from (A and B) showing mean ± SEM from 3 independent experiments (n>25 per experiment). **(D–E)** Recruitment of Src at membrane wraps. Src-YFP expressing cells were infected with RFP expressing Lp for 2h and fixed. Cells were permeabilized and actin stained with phalloidin (green) **(D)** or external bacteria were immunolabeled in un-permeabilized cells (blue; **E**). Higher magnifications of framed regions are shown in panels to the right. **(F)** Src-YFP and PH-Akt-GFP recruitment at membrane wraps. Data shown are means ± SEM from three independent experiments. (n>25 in each experiment). **(G)** Levels of Src (Y416) in cells infected with Lp for the indicated times. Blot shown is representative from 4 independent experiments. Statistical analysis was performed using multiple comparisons one-way ANOVA. **(H)** Lp attachment to cells treated with DMSO or PP2. Cells were treated with DMSO or PP2 for 1h followed by infection with Lp. Attachment was assessed by fluorescence microscopy 1h p.i. Data shown are average number of bacteria attached per cell, normalized to vehicle controls from 4 independent experiments, expressed as a percentage (n>300 cells in each experiment). Error bars shown are SEMs. Statistical analysis was performed using Student's t test. **(I,J)** Recruitment of PH-Akt-GFP in membrane wraps. Cells transiently expressing the probe were infected with RFP expressing Lp for 2 h and fixed, permeabilized and actin stained with phalloidin (green) **(I)** or external bacteria were immunolabeled in un-permeabilized cells (blue; **J**). Panels to the right show higher magnifications of framed regions. **(K)** Levels of Akt (S473) in cells infected with Lp for the indicated times. Blot shown is representative from 3 independent experiments. Statistical analysis was performed using multiple comparisons one-way ANOVA. **(L)** Effect of PI3K inhibition on Lp attachment in cells pre-treated with DMSO or 100 μM Ly294002 (Ly) for 30 min followed by bacterial infection for 1 h. Data shown are mean ± SEM from 3 independent experiments (*n* > 300 cells in each experiment). Statistical analysis was performed using Student's *t*-test. For all fluorescence micrographs the main panels are merged z-stacks and the higher magnifications are single z-planes. All scale bars shown, 12 μm. ns = not significant, ^****^p < 0.0001, ^***^p < 0.001.

The recruitment and activation of β1integrin and E-cadherin receptors at Lp-host cell contact sites trigger the formation of actin-rich membrane wraps that entrap Lp filaments and facilitate their subsequent internalization. As described elsewhere in Prashar et al. (2012) and depicted in Figures 1A–C, these membrane wraps were clearly detectable 2 h after the infection of LECs. To investigate the mechanisms leading to the formation and elongation of membrane wraps, we examined the role of Src and PI3K in these processes. These kinases are critical regulators of actin polymerization downstream of β1integrin and E-cadherin activation (Mitra et al., 2005; Pang et al., 2005; McLachlan et al., 2007). Src was clearly recruited to the membrane wraps and surrounded the segments of the Lp filaments entrapped by these structures, which were inaccessible to external antibodies and therefore, could not be immunolabeled unless the host cells were permeabilized (Figures 1D–F and Figure S1A). The membrane localization of Src indicated its activation by filamentous Lp, which was confirmed by assessing Src (Tyr416) phosphorylation by western blot analysis (Figure 1G). To investigate if Src was required for the attachment of Lp to LECs, we treated cells with the Src family inhibitor PP2 (Hanke et al., 1996) prior to the infection. The average Lp binding to LEC 1 h p.i. was 24.9 ± 2.7 bacteria per 100 cells, determined from 13 experiments. PP2 treatment caused a marked reduction in the attachment of filamentous Lp to the host cells, confirming a role for Src in the invasion process (Figure 1H and Figure S1C). Src acts as an upstream activator of PI3K in several pathways including cadherin based cell-cell adhesions and integrin signaling (Cantrell, 2001; Guo and Giancotti, 2004; McLachlan et al., 2007). Therefore, we next assessed the activity of PI3K in LECs transiently expressing a fusion probe of the pleckstrin homology domain of Akt and GFP (PH-Akt-GFP) that binds to phosphatidylinositol-trisphosphate(Mody et al., 1993; Fields, 1996; Ensminger, 2015), the product of the phosphorylation of phosphatidylinositol-bisphosphate by PI3K (Várnai and Balla, 1998). PH-Akt-GFP was recruited to membrane wraps, indicating that PI3K was active in these structures (Figures 1F,I,J and Figure S1B). Consistent with this, an increase in the levels of Akt activation was observed in cells infected with Lp (Figure 1K). Furthermore, pre-treatment of cells with the PI3K inhibitor Ly294002 (Ly) (Vlahos et al., 1994) reduced the attachment of filamentous Lp to the LECs, therefore indicating a role for PI3K activity in the invasion of LECs (Figure 1L and Figure S1C). Altogether these results demonstrated a role for Src and PI3K in the attachment of filamentous Lp in LECs.

### Rho GTPases and their downstream effectors are required for attachment of filamentous Lp to LECs

Src and PI3K control the phosphorylation states of specific GAPs (GTPase activating proteins), GEFs (guanine nucleotide exchange factors) and GDIs (guanine dissociation inhibitors) to regulate the activation cycle and cellular localization of Rho GTPases, which are central regulators of actin dynamics (Schmidt and Hall, 2002; Hanna and El-Sibai, 2013). Signaling downstream of RhoA activates actin-nucleating formins, while Cdc42 and Rac1 targets include the actin nucleating proteins Arp2/3 (actin related protein) and mDia (Lammers et al., 2008; Hanna and El-Sibai, 2013). Therefore, we sought to investigate if Rho GTPases could be involved in the formation of membrane wraps and the attachment of filamentous Lp in LECs.

**Figure 2:**
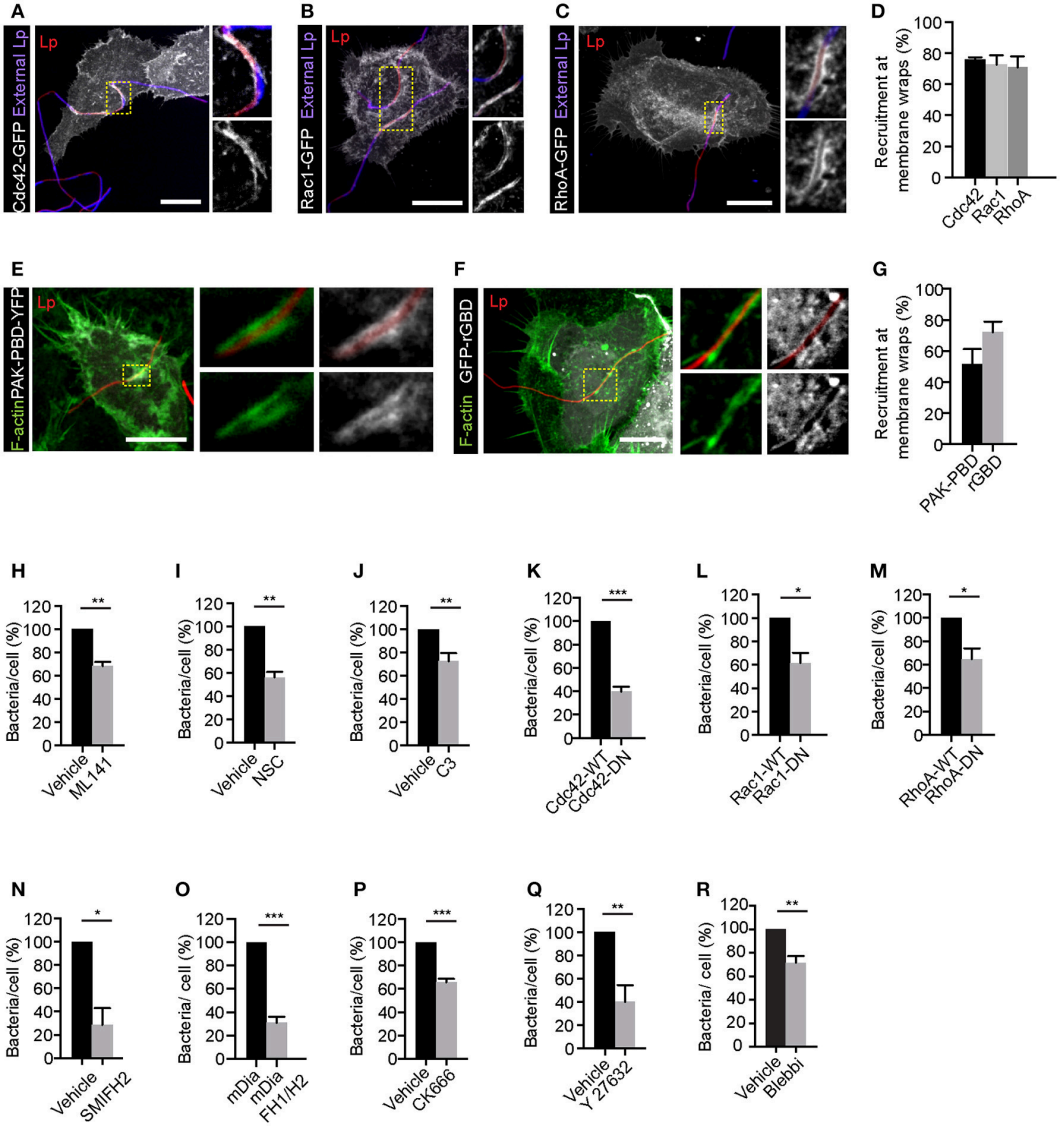
Rho family small GTPases are needed for the attachment of filamentous Lp to LECs. Recruitment of Cdc42 **(A)**, Rac1 **(B)**, and RhoA **(C)** at the membrane wraps formed by Lp attachment. Cells transiently expressing Cdc42-GFP, Rac1-GFP or RhoA-GFP were infected for 2 h, fixed and external bacteria were immunolabeled (blue). Panels to the right show higher magnifications of framed areas. **(D)** Quantification of Rho GTPase recruitment at membrane wraps from **(A–C)**. Means ± SEM from 3 independent experiments are shown (n> 25 bacteria per experiment). **(E,F)** Confocal micrographs showing the recruitment of PAK-PBD-YFP and rGBD-GFP at the membrane wraps formed by Lp 2h p.i. **(G)** Quantification from **(E,F)**. Means ± SEM from 3 independent experiments are shown (*n* > 25 bacteria in each experiment). **(H–J)** Lp attachment to NCI-H292 cells following inhibition of Cdc42, Rac1, and Rho by ML141, Nsc23766 (NSC) and C3 transferase (C3) respectively. Cells were treated with the indicated inhibitors for 1 h followed by infection with Lp for 1 h in the presence of the inhibitors. Quantification of bacterial attachment, normalized to vehicle treated cells is shown. **(K–M)** Attachment of Lp to cells expressing wild-type or dominant-negative (DN) forms of Rho GTPases. Attachment of bacteria to cells pre-treated for 1 h with inhibitor for mDia **(N)** or cells expressing dominant negative form of mDia (mDia-DN) **(O)**. Attachment of bacteria to cells pre-treated with CK666, Y27632 or blebbistatin to inhibit Arp2/3, ROCK or myosin II respectively **(P–R)**. Data shown are means ± SEM where bacterial attachment to at least 200 cells was analyzed. The number of independent experiments analyzed for each treatment were as follows: **(H,I,K–P,R)** *n* = 3; **(J,Q)** *n* = 4. Vehicle controls for each condition were as follows: **(H,N–R)** DMSO; **(I)** water; **(J)** glycerol. For all fluorescence micrographs the main panels are merged z-stacks and the higher magnifications are single z-planes. All scale bars shown, 12 μm. Statistical tests were performed using Student's *t*-test, ^***^p < 0.0001, ^**^p < 0.001, ^*^p < 0.05.

As shown in Figures 2A–D and Figures S2A–C, Rho GTPases Cdc42, Rac1 and RhoA were recruited to the membrane wraps. Furthermore, PAK-PBD (p21 activated kinase binding domain) and rGBD (Rho binding domain of Rhotekin), which bind to activated Cdc42/Rac1 and Rho respectively (Burbelo et al., 1995; Ren et al., 1999), were also recruited to the membrane wraps (Figures 2E–G and Figure S2D,E), indicating that these Rho GTPases were activated at the site of Lp attachment and membrane wrap formation. Accordingly, both the pre-treatment of cells with any one of the Cdc42, Rac1 and Rho inhibitors (Figures 2H–J) or the expression of dominant negative alleles for these Rho GTPases (Figures 2K–M), caused a significant decrease in the attachment of filamentous Lp to LECs. Thus, we concluded that Rho GTPases were needed for the attachment of filamentous Lp to the LECs.

We next investigated if the actin-nucleating proteins downstream of Rho GTPase were contributing to filamentous Lp attachment. Consistent with this possibility, inhibiting the actin nucleating activity of the formin mDia prior to infection with SMIFH2 or by expressing the dominant negative construct FH1/H2, inhibited the attachment of filamentous Lp to LECs (Figures 2N,O). Pre-treatment of cells with CK666 to inhibit Arp2/3 activity also caused a reduction in bacterial attachment (Figure 2P). Altogether these results indicated that actin polymerization into both linear and branched actin filaments, associated with the formation of filopodia and lamellopodia (Goley and Welch, 2006; Goode and Eck, 2007), respectively, is required for the initial attachment of Lp to LECs.

In addition to controlling the actin nucleators, Rho activity can also regulate actin-myosin contractility by controlling myosin II activity via the Rho effector ROCK (Rho-associated protein kinase) (Hanna and El-Sibai, 2013). Myosin II-dependent actin bundling and contraction have been previously shown to be required for membrane wrap elongation and maintenance (Prashar et al., 2012). Interestingly, treatment of LECs with specific ROCK or myosin II inhibitors Y27632 and blebbistatin, respectively also caused a reduction in filamentous Lp attachment to LECs (Figures 2Q,R), suggesting the potential involvement of myosin II-dependent actin contraction in the entrapment of filamentous Lp by closing the membrane wraps to secure filamentous Lp at the LEC surface.

Collectively, these results indicated that the activation of Rho GTPases and their downstream actin nucleating proteins were responsible for the changes in actin polymerization and contractility required for the membrane wrap-mediated attachment of the filamentous bacteria to the LEC surface.

### Rho GTPases are needed for the elongation of membrane wraps and internalization of filamentous Lp in LECs

**Figure 3:**
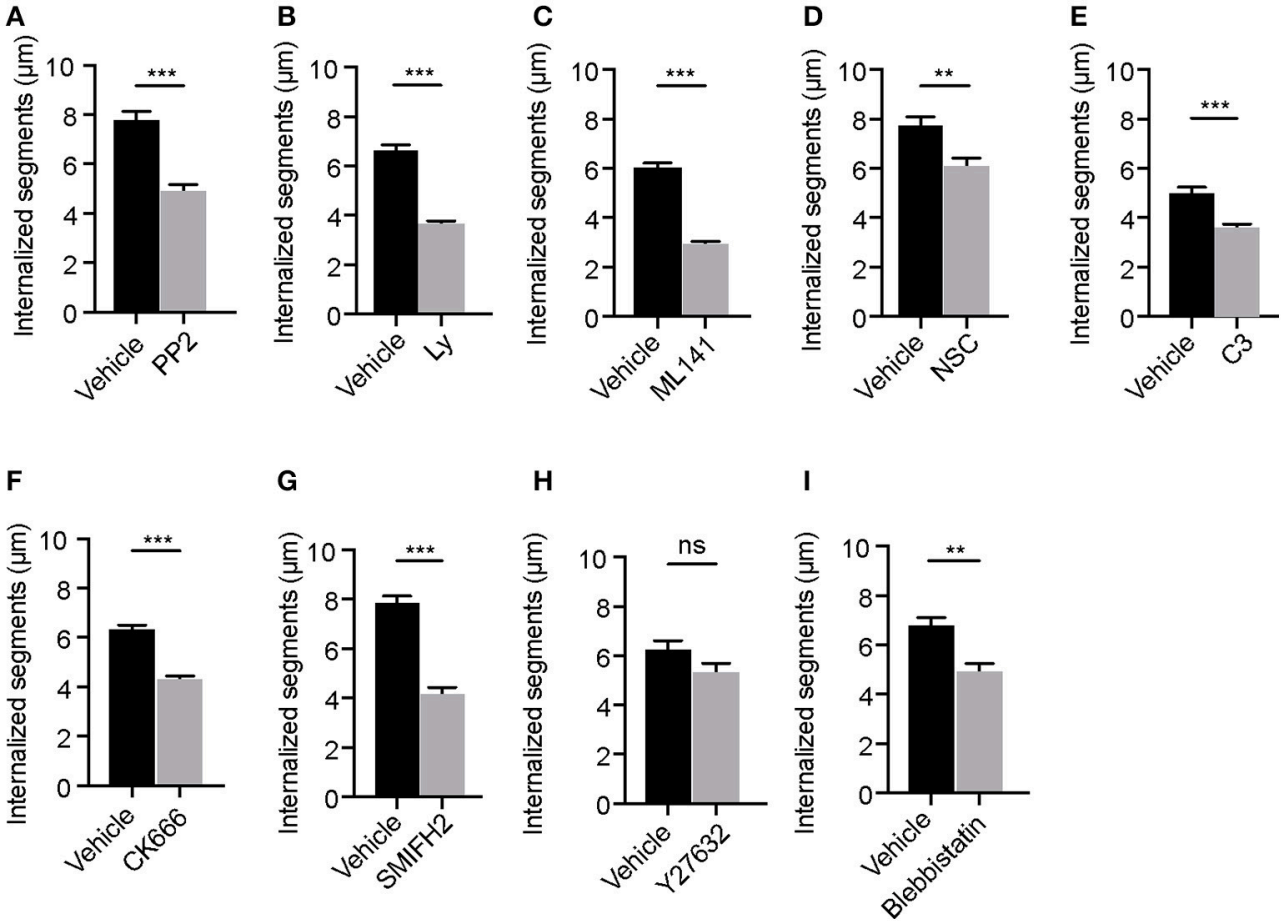
Elongation of membrane wraps requires the activity of Src and PI3 kinases and Rho GTPases. Quantifications of the lengths of the segments of Lp filaments internalized in cells following the indicated treatments: **(A)** DMSO or Src inhibition with PP2, **(B)** DMSO or PI3K inhibition with Ly294002 (Ly), **(C)** DMSO or Cdc42 inhibition with ML141, **(D)** Water or Rac1 inhibition with NSC23766 (NSC), **(E)** Glycerol or Rho inhibition using C3 transferase (C3), **(F)** DMSO or Arp2/3 inhibition using CK666, **(G)** DMSO or mDia inhibition using SMIFH2, **(H)** DMSO or ROCK inhibition using Y27632 and **(I)** DMSO or Myosin II inhibition using blebbistatin. Cells were infected with RFP-Lp for 1h. Unbound bacteria were washed and the indicated inhibitors were added for 5 h before cells were fixed and external bacteria were immune-labeled. Internalization was quantified by measuring the lengths of the Lp segments that were inaccessible to the external antibody applied in non-permeabilized cells (see materials and methods). Data shown are mean ± SEMs from 3 independent experiments. Each segment of an Lp filament undergoing internalization was considered as an independent value. At least 100 filaments were analyzed in each condition. Statistical analyses were performed using Student's *t*-test, ^***^p < 0.0001, ^**^p < 0.001, ^*^p < 0.05.

Our previous findings using synchronized infections showed that internalization of filamentous Lp by LECs requires the elongation of membrane wraps (Prashar et al., 2012). Individual membrane wraps elongate over time and merge with each other, internalizing longer segments of Lp filaments in an actin-rich cradle (Prashar et al., 2012). Considering this, we reasoned that the internalization of Lp filaments would require membrane and actin cytoskeleton rearrangements, probably controlled by the same signaling axes discussed above. Thus, we investigated the role of Src, PI3K and Rho GTPases in the elongation of membrane wraps by measuring the length of the Lp segments entrapped by these structures. To this end, LECs were infected with RFP-Lp for 1 h to allow membrane wraps to form, and subsequently treated with specific inhibitors for Src, PI3K or Rho GTPases for an additional 5 h period. As shown in Figures 3A–G,I, inhibiting the activity of Src, PI3K, Rho GTPases or their downstream effectors except for the RhoA downstream effector ROCK, caused a reduction in the length of the bacterial segments internalized in LECs. Taken together, these findings supported a role for Rho GTPases in the elongation of membrane wraps, leading to the internalization of filamentous Lp in LECs.

### Contribution of Dot/Icm type IV secretion system (T4SS) to Lp attachment and internalization in LECs

Bacterial Invasion of non-phagocytic cells is a pathogen driven process that requires the coordinated activity of adhesins, toxins and effectors (Cossart and Sansonetti, 2004). Even though the phagocytic uptake of Lp T4SS mutants (*dotA* mutants) by macrophages has been shown to be defective (Hilbi et al., 2001), the role of T4SS-delivered effectors in attachment and entry of Lp in LECs is not known.

**Figure 4:**
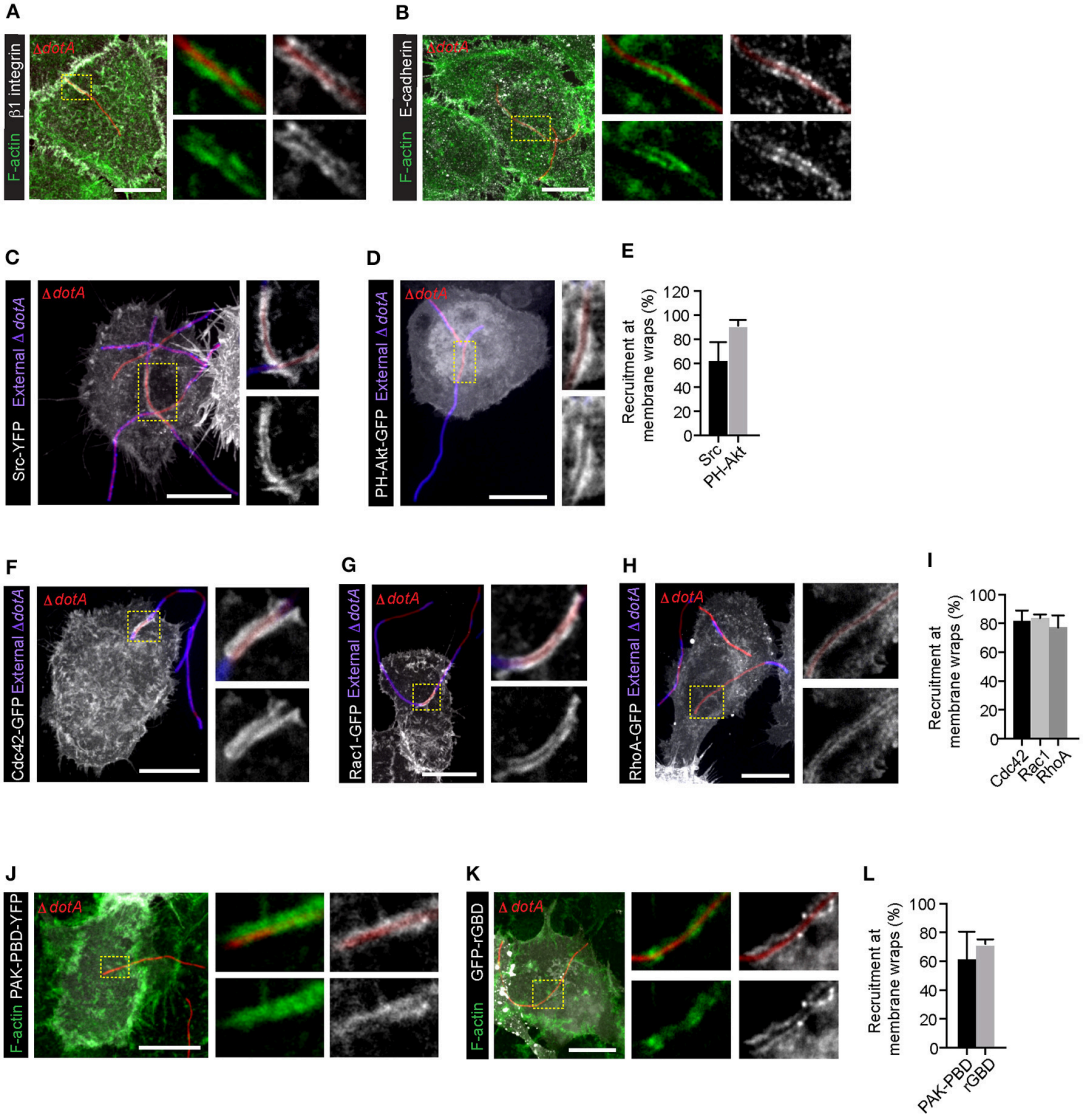
β1 integrin, E-cadherin receptors and downstream signaling molecules are recruited to the membrane wraps independently of Dot/Icm translocated effectors. Representative confocal micrographs showing the recruitment of β1integrin **(A)** and E-cadherin **(B)** receptors in membrane wraps formed by RFP expressing *dotA* mutants 2 h p.i. Green:F-actin. Higher magnifications of areas indicated by dashed lines are in panels to the right. **(C,D)** Recruitment of Src and PH-AKT (grayscale) in cells infected with *dotA* mutants for 2 h. Blue: external bacteria. Panels to the right show higher magnification of the indicated regions. **(E)** Quantification from **(C,D)**. Data shown are mean ± SEM from 3 independent experiments (*n* > 25 filaments per experiment). **(F–H)** Confocal micrographs showing the recruitment of Rho GTPases to sites of attachment of *dotA* mutants. Cells expressing Cdc42-GFP **(F)**, Rac1-GFP **(G)** or RhoA-GFP **(H)** were infected with RFP-*dotA* mutants for 2 h, fixed and external bacteria were immunolabeled using anti-Lp antibodies (blue). Panels to the right show higher magnifications of the areas indicated in the main panels. **(I)** Quantification from **(F–H)**. Data shown are means ± SEM from 3 independent experiments (*n* > 25 filaments in each experiment). **(J,K)** Confocal micrographs showing the recruitment of PAK-PBD-YFP **(J)** and rGBD-GFP **(K)** at the membrane wraps formed by RFP-*dotA* 2 h p.i. Panels to the right show higher magnifications of indicated regions. **(L)** Quantification from **(J,K)**. Data shown are mean ± SEM from 3 independent experiments (*n* > 25 bacteria in each case). For all fluorescence micrographs the main panels are merged z-stacks and the higher magnifications are single z-planes. All scale bars, 12 μm.

**Figure 5:**
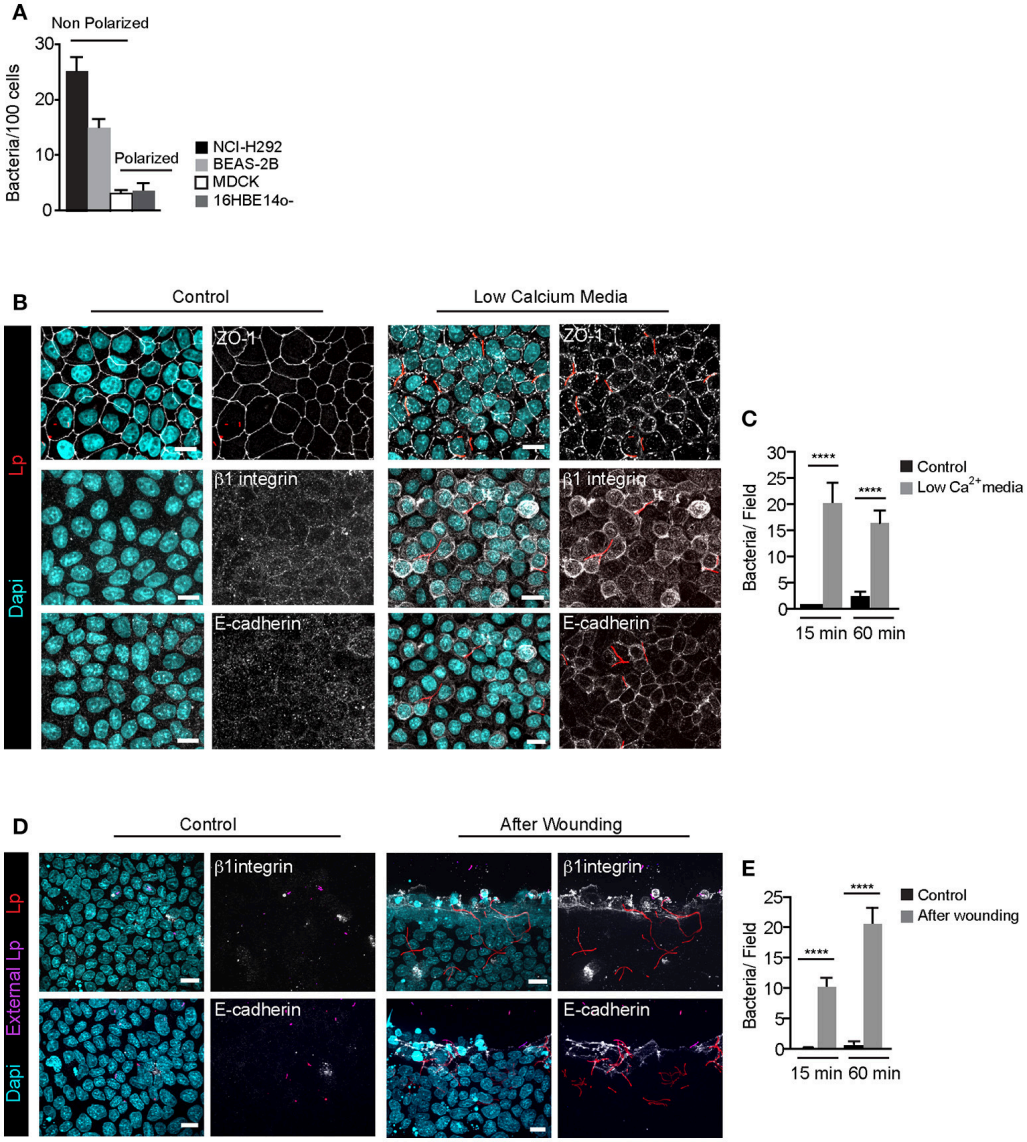
Filamentous Lp do not attach to polarized epithelial cell monolayers **(A)** Efficiency of Lp attachment to different LECs 1 h p.i. Data shown are means±SEM from 2 independent experiments. Attachment to at least 200 cells was quantified in each experiment. **(B)** Effect of low calcium media on tight junction permeability. MDCK cell monolayers treated with low calcium media (see Materials and Methods) were infected with RFP-Lp for 15 or 60 min, fixed and external bacteria were immunolabeled (purple). Cells were permeabilized and immunolabeled using anti-Zo1 antibody (top panels), anti-β1 integrin (middle panels) or anti-E-cadherin antibodies (bottom panels). Panels to the left show uninfected control cells. Dapi: cyan. **(C)** Quantification of Lp attachment to MDCK monolayers after treatment with low calcium media. Data shown are mean ± SEMs from 3 independent experiments. **(D)** Lp attachment to MDCK cell monolayers after mechanical disruption of tight junctions. Monolayers were wounded and infected with RFP-Lp for 10 or 60 min and fixed. External bacteria and β1 integrin or E-cadherin receptors (grayscale) were immunolabeled in unpermeabilized cells. **(E)** Quantification of bacterial attachment from **(D)**. Data shown are mean ± SEM from 3 independent experiments. All scale bars, 12 μm. Statistical analysis was performed using Student's *t*-test, ^****^p < 0.0001.

We previously showed that Lp *dotA* mutants can attach to the LECs and form F-actin rich membrane wraps (Prashar et al., 2012). However, whether these membrane wraps formed by *dotA* mutants elongate to cause bacterial internalization, similar to wild-type bacteria, was not assessed. To investigate this, we first assessed the intracellular signaling triggered by *dotA* mutants at the surface of NCI-H292 cells. As shown in Figures 4A,B, both β1integrin and E-cadherin receptors were recruited to the actin-rich membrane wraps formed by *dotA* mutants with an efficiency of 92 and 89% respectively, and also similar to wild-type Lp (as shown in Figures 1, 2), Src, PH-Akt and Rho GTPases were also recruited to these membrane wraps (Figures 4C–L). Therefore, these results indicated that the formation of membrane wraps occurred independently of T4SS effectors. Rather, it was the activation of host receptors, and the subsequent downstream signaling that was needed for their formation. This is in agreement with our previously reported findings that blocking or silencing the expression of β1 integrin and E-cadherin receptors was sufficient to inhibit the attachment of wild-type Lp to LECs (Prashar et al., 2012). To further confirm that attachment to receptors was solely responsible for bacterial binding to LECs, we assessed the ability of Lp to attach and invade polarized epithelial cells, where β1integrin and E-cadherin receptors are segregated to the basolateral surface of the cell (Pentecost et al., 2006). Lp was unable to attach to polarized cell monolayers (Figure 5A). However, Lp attachment to MDCK (Madin-Darby Canine Kidney) cells increased when tight junctions were disassembled in low calcium media (Figures 5B,C and Figure S3) or by wounding the monolayers (Figures 5D,E), exposing β1integrin and E-cadherin receptors. Collectively, these results demonstrated that the binding to host cell receptors, and not T4SS effectors, led to the formation of primordial membrane wraps necessary for the first step in the attachment of Lp to LEC.

**Figure 6:**
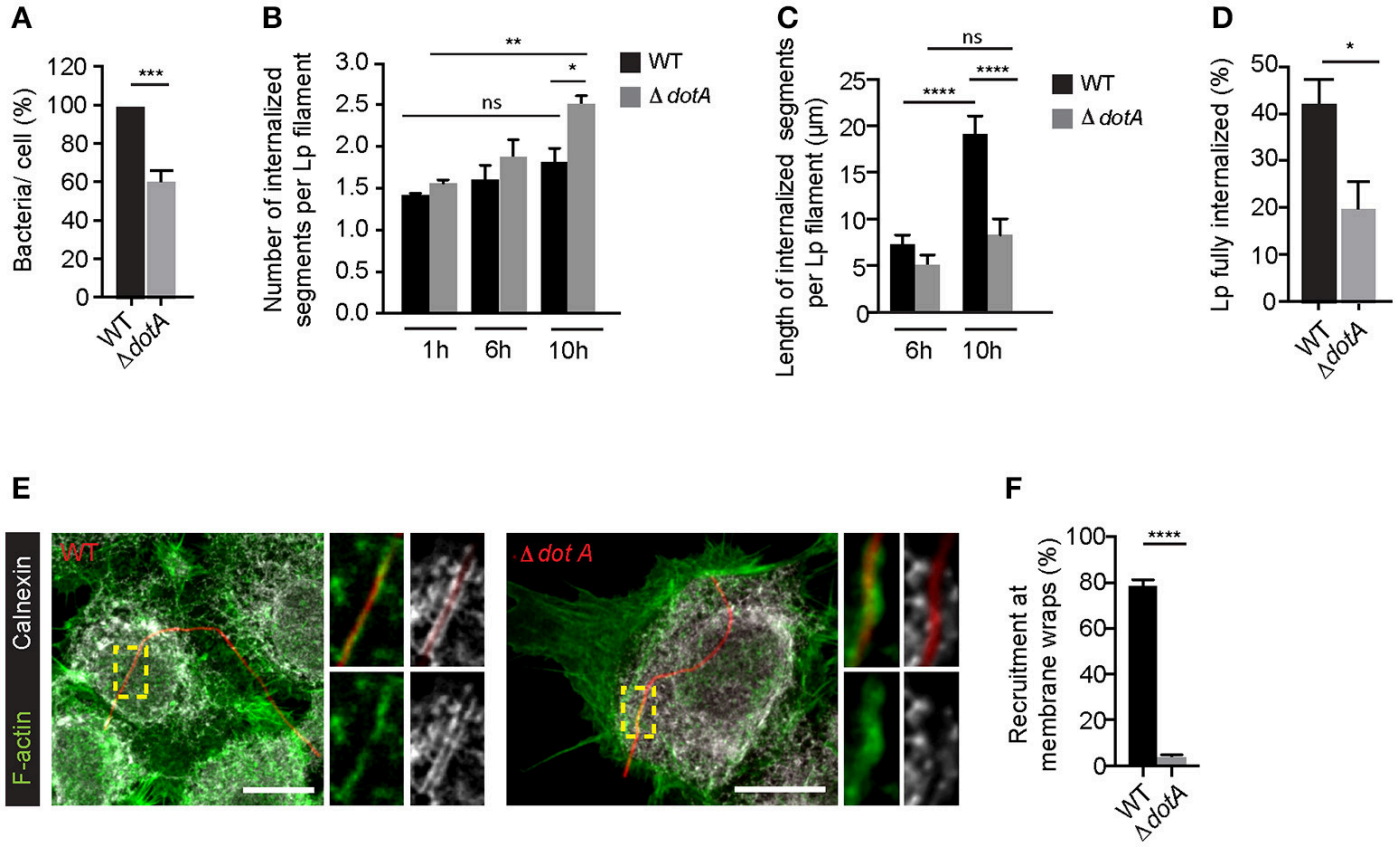
Dot/Icm translocated effectors are required for the attachment and entry of filamentous Lp in LECs **(A)** Quantification of the efficiency of attachment of *dotA* mutants compared to wild-type Lp 1 h p.i. Data shown are mean ± SEM from 5 independent experiments, normalized to wild-type controls (*n* > 500 cells per experiment). Statistical analysis was performed using Student's. ^***^p < 0.001. **(B)** Number of internal segments for wild-type and *dotA* mutants. Cells infected with RFP-WT or RFP-*dotA* mutants were washed at 1 h p.i to remove unbound bacteria and fixed or internalization was allowed to proceed for additional 5 or 9 h followed by immunolabelling of external bacteria. Each segment undergoing internalization was considered as a separate event. Data shown are mean ± SEM from 3 independent experiments (*n* > 100 in each experiment). Statistical tests were performed using one way- ANOVA with Tukey's multiple comparison test, ^*^p < 0.05. **(C)** Internalization of *dotA* mutants compared to wild-type Lp 6 and 10 h p.i. Lengths of bacteria internalized were measured using differential immunolabeling to label external bacteria. Data shown are mean ± SEMs from 3 independent experiments (*n* > 100 bacteria in each experiment). Statistical tests were performed using one way- ANOVA with Tukey's multiple comparisons test, ^****^p < 0.0001. **(D)** Efficiency of bacterial internalization at 12 h p.i. Cells infected with RFP-WT or RFP-*dotA* mutants were washed 1 h p.i. to remove unbound bacteria. Cells were fixed 12 h p.i and external bacteria were immunolabeled. The number of bacteria fully internalized was quantified in three individual experiments. Data shown are mean ± SEM from 3 independent experiments (n>100 in each experiment). Statistical analysis was performed using Student's, ^*^p < 0.01. **(E)** Representative confocal micrographs showing the recruitment of calnexin (grayscale) and F-actin (green) at the membrane wraps formed by attachment of RFP-WT (left) or RFP-*dotA* mutants (right) 6 h p.i. For all fluorescence micrographs the main panels are merged z-stacks and the higher magnifications are single z-planes. All scale bars, 12 μm. **(F)** Quantification from **(E)**. Data shown are means± SEMs from 3 independent experiments (*n* > 25 in each case). Statistical analysis was performed using Student's *t*-test, ^****^p < 0.0001.

Interestingly, while *dotA* mutants were able to attach to LECs and form membrane wraps, the attachment occurred with a lower efficiency (Figure 6A). Furthermore, we detected differences in how the subsequent steps in their internalization proceeded when compared to wild-type Lp. As the infection progressed, there were a greater number of internalized segments per bacteria filament (i.e., number of membrane wraps) produced by *dotA* mutants compared to wild-type Lp. However, the length of these internalized segments for *dotA* was significantly shorter than those for wild-type bacteria (Figures 6B,C). The fact that *dotA* mutants produced a greater number of internalized segments, but of a shorter length, suggested that the membrane wraps formed by the mutant strain failed to efficiently elongate and/or merge to internalize longer bacterial segments.

Supporting this notion, at later stages of infection, *dotA* mutants were less efficient in entering LECs, as indicated by the number of bacteria completely internalized at 12 h p.i (Figure 6D). Importantly, this reduction in the internalization of *dotA* mutants was not due to differences in the lengths of the two strains (Figure S4). Thus, collectively our results strongly suggest that *dotA* translocated effectors may contribute to the internalization of filaments by LECs. If T4SS translocated effectors contribute to the internalization of filaments by LECs, these effectors must be delivered into the host cells at the sites of Lp attachment, prior to the filaments being fully internalized. Indeed, 6 h p.i, calnexin, a *bona fide* marker of LCVs that is recruited to these compartments when Lp T4SS effectors hijack the host ER to Golgi vesicular trafficking pathway (Isaac and Isberg, 2014), was recruited by wild-type but not *dotA* mutants to the sites of bacterial attachment (Figures 6E,F).

### T4SS translocated effector VipA contributes to Lp attachment and entry in lung epithelial cells

**Figure 7:**
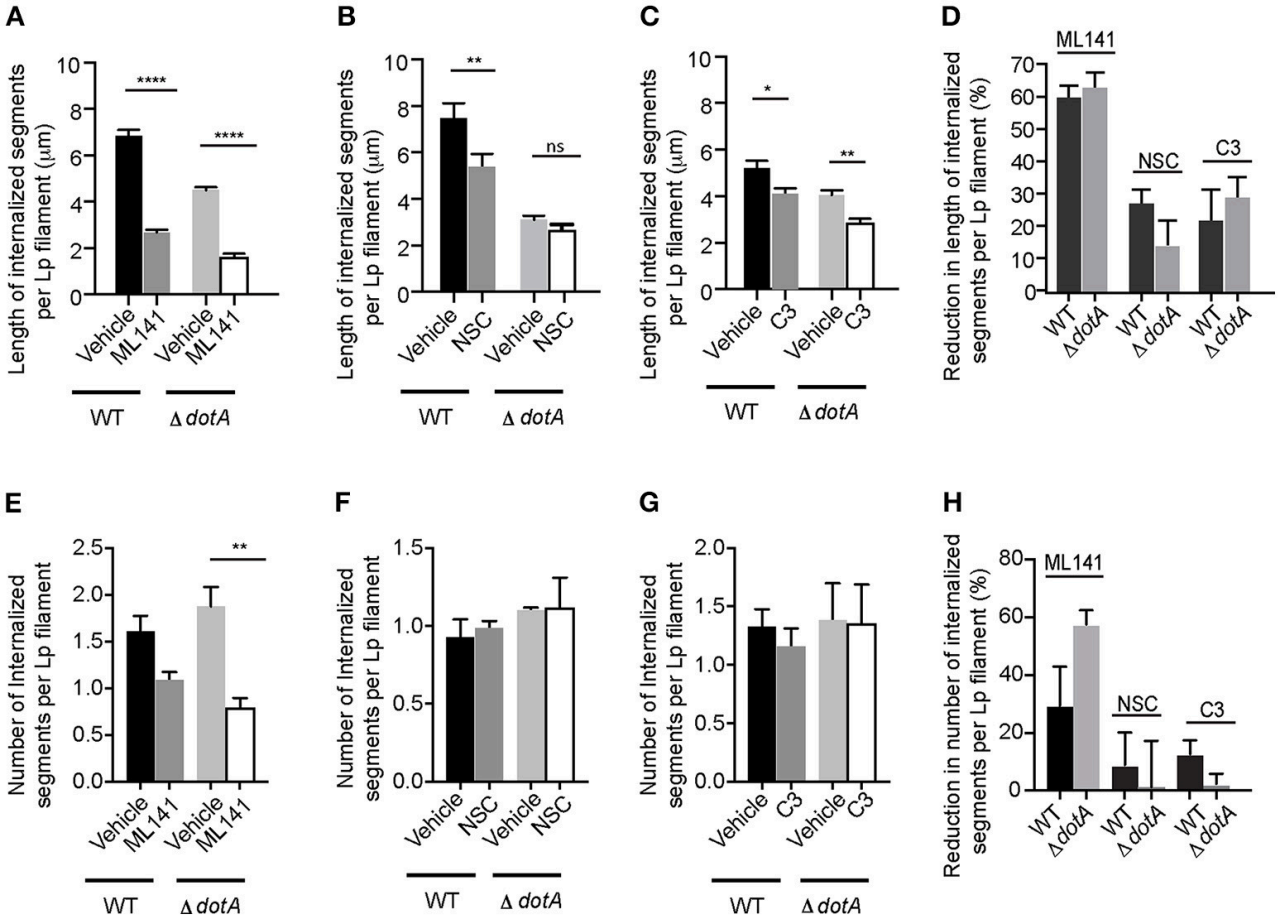
Rho GTPases and T4SS act independently during the internalization of filamentous Lp in LECs **(A–C)** Effect of Rho GTPase inhibition on the internalization efficiency of *dotA* mutants. Cells were infected with RFP-WT or RFP- *dotA* mutants for 1 h. Unbound bacteria were washed and the indicated inhibitors were added. Internalization was allowed to proceed for 5 h in the presence of the inhibitors. Cells were then fixed and external bacteria were immunolabeled. Lengths of bacteria internalized 6 h p.i. were measured based on the inaccessibility of external antibody applied prior to permeabilization. Data shows are mean ± SEM from 3 independent experiments. **(D)** Decrease in Lp internalization from **(A–C)** relative to respective vehicle controls. **(E–G)** Number of internal segments of Lp filament from **(A–C)**. **(H)** Decrease in the number of Lp segments undergoing internalization via membrane wraps from **(E–G)** relative to their respective vehicle controls. At least 100 bacteria were analyzed in each experiment. Statistical analysis was performed using one-way ANOVA with Tukey's multiple comparisons test. ^****^p < 0.0001, ^***^p < 0.001, ^**^p < 0.01, ^*^p < 0.05.

Pathogenic bacteria can subvert host cell actin-cytoskeleton through effectors that co-opt Rho GTPases (Guttman and Finlay, 2009; Kim et al., 2010; Bonazzi and Cossart, 2011). However, *dotA* mutants were able to recruit and activate Rho GTPases in the membrane wraps (Figure 4) and there were no differences in the effect of inhibiting the activity of Rho GTPases on the elongation (Figures 7A–D) or number (Figures 7E–H) of membrane wraps formed by both wild-type Lp or *dotA* mutants. Therefore, we discarded the possibility that Lp T4SS-translocated effectors could contribute to the elongation of membrane wraps via Rho-GTPases.

**Figure 8:**
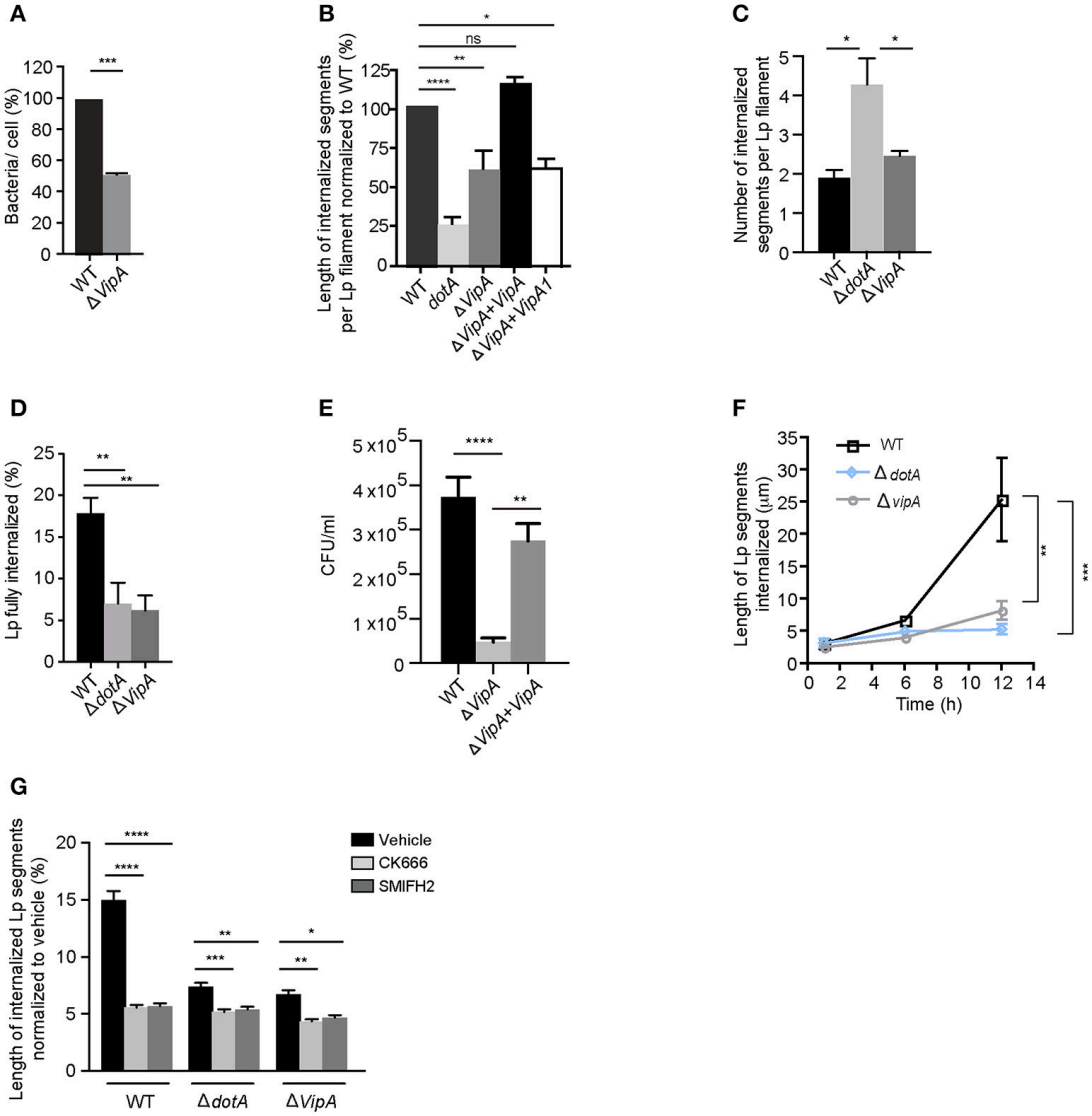
Lp effector VipA mediates Lp attachment and entry in LECs **(A)** Attachment efficiency of *vipA* deletion mutants compared to wild-type Lp. Cells were fixed 1 h p.i and attachment was quantified. Data shown are mean ± SEM from 3 independent experiments, normalized to wild-type as 100%. (*n* > 100 cells in each experiment). Statistical analysis was performed using Student's *t*-test ^***^p < 0.001. **(B)** Internalization efficiency of indicated Lp strains 10 h p.i. Cells were infected for 1 h, washed and infections were allowed to proceed for an additional 9 h. Cells were fixed and external bacteria were immunolabeled using anti-Lp antibodies followed by cell permeabilization and immunolabeling of total bacteria. Internalization was measured using antibody exclusion prior to internalization. Data shown are mean ± SEM from 3 independent experiments, relative to the length of wild-type Lp, expressed as 100% (*n* > 100 bacteria in each experiment). **(C)** Number of membrane wraps formed by the indicated Lp strains 12 h p.i. Data shown are mean ± SEMs from 3 independent experiments (*n* > 50 bacteria in each case). **(D)** Lp filaments fully internalized by LECs 12 h p.i. Data shown are mean ± SEM from 3 independent experiments (*n* > 30 in each case). **(E)** CFU counts showing wild-type Lp, *dotA* or *vipA* mutants internalized in LECs 18 h p.i (see Materials and Methods). Data shown are mean ± SEMs from 5 independent experiments. **(F)** Internalization efficiency of wild-type Lp compared to *dotA* mutants and *vipA* deletion mutants at the indicated times. Infected cells were washed at 1 h p.i to remove unbound bacteria and cells were fixed at 1, 6, or 12 h p.i. External bacteria were labeled using anti-Lp antibodies, followed by permeabilization of cells and total bacteria were labeled. Exclusion of external antibody prior to permeabilization was used to measure the length of bacteria undergoing internalization at each time point. Data shown are mean ± SEM from 3 independent experiments (*n* > 150 bacteria in each experiment). **(G)** Effect of inhibiting actin nucleators on internalization of wild-type Lp, *dotA* or *vipA* mutants analyzed 12 h p.i. Cells were infected for 1 h, washed and treated with DMSO or the indicated inhibitors for 11 h before fixation. External bacterial were immunolabeled and lengths of Lp segments undergoing internalization were measured. For all fluorescence micrographs the main panels are merged z-stacks and the higher magnifications are single z-planes. All scale bars, 12 μm. Unless stated otherwise, all statistical analyses were performed using one-way ANOVA with Tukey's multiple comparison test, ^****^p < 0.0001, ^***^p < 0.001, ^**^p < 0.01, ^*^p < 0.05.

How do T4SS effectors contribute to the attachment and internalization of filamentous Lp in LECs? Our findings from Figure 6 showing that both *dotA* mutants and wild-type Lp generated a similar number of membrane wraps early during infection, confirmed that T4SS effectors were not required for the formation of the primordial wraps. However, the membrane wraps generated by *dotA* mutants were shorter compared to wild-type Lp, indicating that a bacterial encoded effector could instead be a key contributor in the elongation and/or merging of individual membrane wraps. To investigate this possibility we assessed the role of the Lp T4SS effector VipA that has been shown to bind actin and enhance actin polymerization *in vitro* by acting as a G-actin nucleating protein (Franco et al., 2012). To this end, we generated a *vipA* deletion mutant to examine the contribution of this effector toward the internalization of Lp filaments in LECs (Figures S5A,B). Similar to *dotA* mutants (Figure 6A), *vipA* mutants showed a reduction in the number of bacteria attached to LECs 1 h p.i. when compared to wild-type Lp (Figure 8A). The internalized bacterial segments generated by *vipA* mutants (i.e., membrane wraps) were shorter, but more numerous, when compared to wild-type bacteria (Figures 8B,C). This phenotype was rescued to wild type levels by reintroducing a *vipA* allele but not with the *vipA*-1 mutant allele, which is defective in actin nucleating activity (Shohdy et al., 2005; Franco et al., 2012) in the *vipA* mutant strains (Figure 8B). Accordingly, *vipA* mutants, with lengths comparable to wild-type Lp (Figure S5C), were impaired in their ability to enter LECs, as assessed by the numbers of fully internalized bacteria, and consequently produced less intracellular progeny (Figures 8D,E and Figure S5D).

**Figure 9:**
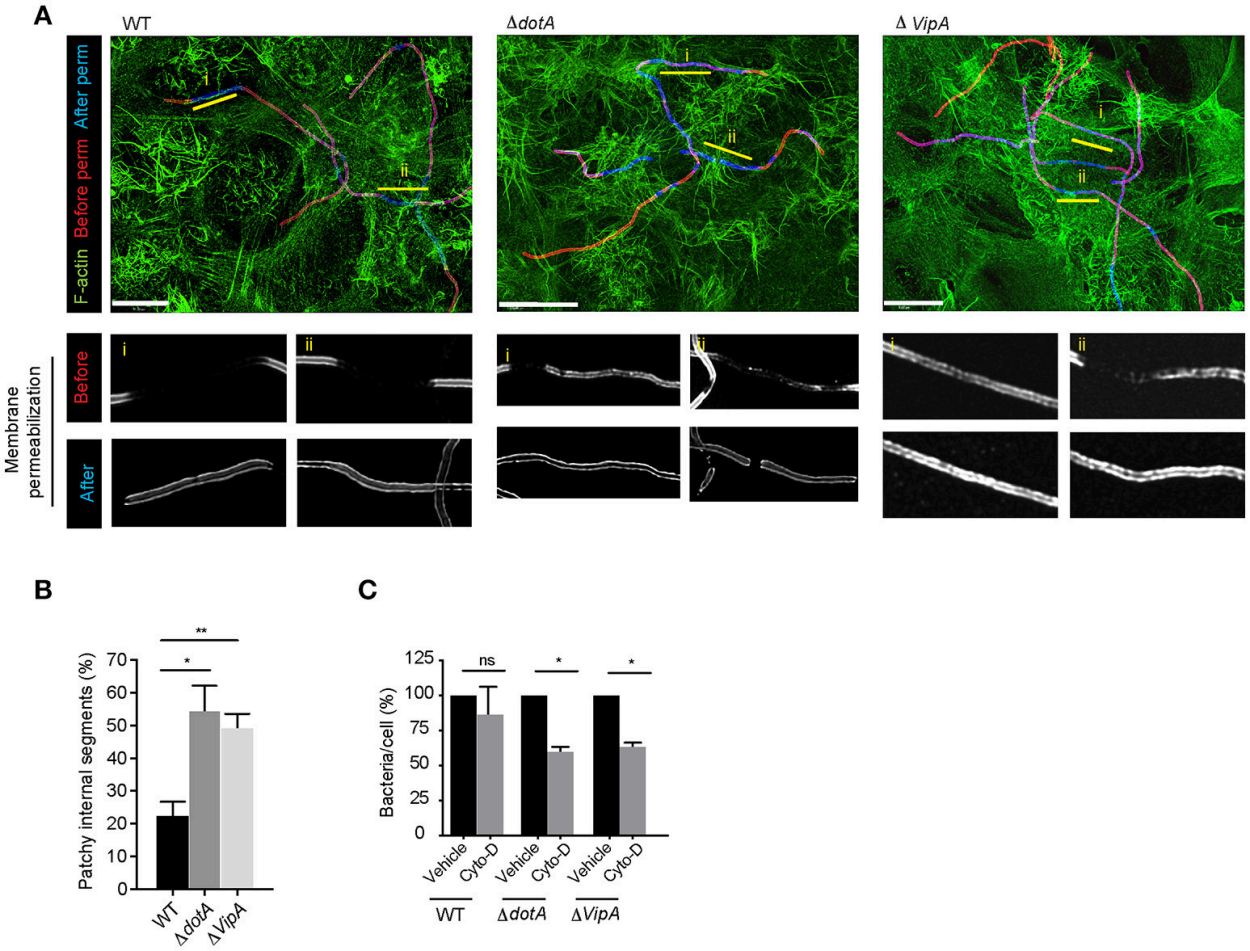
VipA contributes to membrane wrap formation **(A)** Representative deconvolved confocal micrographs showing the immuno-labeling pattern depicting the defective barrier formation at membrane wraps formed by *dotA* and *vipA* mutants compared to wild-type Lp. LECs were infected for 1 h, washed and fixed 6 h p.i. External bacteria were immunolabeled (red), cells were permeabilized and internal bacteria were immunolabeled using anti- Lp antibodies (blue) and F-actin was stained using phalloidin (green). **(B)** Quantification from **(A)**. Data shown are mean ± SEM from 3 independent experiments (*n*>80 filaments in each experiment). **(C)** Lp attachment to LECs following treatment with cytochalasin-D. Bacterial attachment to vehicle treated controls has been normalized to 100% and attachment following treatment is expressed as relative values. Data shown are mean ± SEM from 3 independent experiments (n>1000 cells in each experiment). For all fluorescence micrographs the main panels are merged z-stacks and the higher magnifications are single z-planes. All scale bars, 12 μm. Statistical analyses were performed using one-way ANOVA with Tukey's multiple comparison test. ^**^p < 0.01, ^*^p < 0.05.

Assessing the length of internalized segments overtime for wild-type, *dotA* and *vipA* mutants, we found that while the length of the internalized segments ramped to 25.4 μm at 12 h p.i for wild-type Lp, it stalled for both *dotA* and *vipA* mutants (Figure 8F). Furthermore, while there were small differences in internalization between *dotA* and *vipA* mutants, the lack of VipA activity alone was largely sufficient to account for the defect in internalization observed with *dotA* mutants (Figures 8D–F). Thus, the actin nucleating activity of VipA may be responsible for the elongation of individual membrane wraps that will ultimately merge with their neighbors, allowing for the internalization of longer segment of bacteria, leading to their fully internalization by LECs. Accordingly, blocking the formation of new membrane wraps by treating the infected cells with Arp2/3 and mDia inhibitors 1 h p.i, produced shorter internalized segments (i.e. membrane wraps), which was accompanied by a reduction in their number (Figure S5E) at 12 h p.i. Therefore both, VipA and the cellular actin-nucleating proteins contributed to the elongation of membrane wraps. However, the actin-nucleating inhibitors only caused minor reduction in the length of the already shorter membrane wraps produced by *dotA* and *vipA* mutants, assessed at 12 h p.i (Figures 8F,G). Thus, the contribution of the cellular-actin nucleating proteins to the elongation of membrane wraps, beyond the formation of the primordial structure may be conditioned by VipA activity. Considering this, we speculated that the elongation of a primordial membrane wrap by VipA could favor the close contact of the attached Lp filament against the LEC surface nearby the original membrane wrap. At these sites, the activation of β1integrin and E-cadherin receptors could cause new membrane wraps to form and elongate to eventually merge with the older wraps. Supporting a role of VipA in the morphogenesis of membrane wraps, we detected structural and functional changes in the membrane wraps associated with a lack of VipA expression. The membrane wraps formed by both *vipA* and *dotA* mutants, assessed at 6 h pi, were defective in the barriers required to prevent the antibodies applied in un-permeabilized cells from diffusing and immunolabelling internalized bacterial segments, rendering a “patchy” staining phenotype (Figures 9A,B).

Given the defective barriers at the membrane wraps formed by the mutant strains, we surmised that the “patchy” phenotype could reflect defects in the development of membrane wraps which would have consequences for how securely these structures trapped Lp filaments at the cell surface. We previously showed that the disruption of the membrane wraps with cytochalasin-D during the early stages of their morphogenesis causes the release of the entrapped Lp filaments and their detachment from the LECs (Prashar et al., 2012). Similarly, a short pulse with cytochalasin-D, even at 6 h p.i. caused the detachment of both *vipA* and *dotA* mutants, while wild-type Lp were not affected (Figure 9C), demonstrating the need for VipA in membrane wrap morphogenesis.

Altogether, these findings support the role of the T4SS translocated effector VipA in promoting Lp attachment to and entry of LECs, through its role in facilitating actin polymerization needed for membrane wrap formation and elongation over entrapped Lp filaments.

## Discussion

Stressful environmental stimuli like host effectors, protist predation or antimicrobial treatments can lead to bacterial filamentation, and this morphology can provide the bacteria with a survival advantage (Justice et al., 2008). The process of invasion of host cells by filamentous bacteria has only been reported for the urinary tract pathogen *Proteus mirabilis* (Allison et al., 1992) and long chains of *Streptococcus* (Molinari et al., 2000), however the invasion mechanisms remain unknown. We previously showed that filamentous Lp is more efficient than rods in attaching to LECs and that the invasion of LECs by filamentous Lp requires the engagement of β1 integrin and E-cadherin receptors at the host cell surface (Prashar et al., 2012). Here we demonstrate that the binding to these host cell receptors causes the downstream activation of Src and class I PI3 kinases, and members of the Rho family of small GTPases, namely Cdc42, Rac1, and RhoA. These in turn induce actin polymerization via the actin nucleating proteins Arp2/3 and formins (mDia). We show that these pathways are critical for the initial attachment of Lp filaments and the formation and elongation of the primordial membrane wraps that prime the internalization of Lp filaments. While further work is required to dissect the hierarchy and individual contribution of each one of the small GTPases toward the morphogenesis of membrane wraps and internalization of Lp, our results indicate a more preponderant contribution of the Cdc42-mDia axis to these processes. Cdc42-mDia have been previously associated with the formation of filopodia (Mattila and Lappalainen, 2008; Mellor, 2010) and hence may control the formation of hooks required for the initial attachment of Lp, and also contribute to the extension of the leading edges of lamellas that form the membrane in wraps.

Along with actin polymerization, RhoA also modulates the forces exerted by the actin cytoskeleton in filopodia and lamellopodia by regulating the actin motor myosin II (Sit and Manser, 2011; Sayyad et al., 2015). Accordingly, we found that myosin II plays a role in the formation of membrane wraps, potentially by providing the force required to close the opposing lamellar protrusions that form these structures. Supporting this, blebbistatin (Straight et al., 2003) inhibits membrane wrap formation and elongation. Rho-associated protein kinase (ROCK) activates myosin II activity via the phosphorylation of myosin light chain (MLC) (Kimura et al., 1996). Despite the requirement for myosin II in Lp attachment and internalization, ROCK inhibition with Y27632 (Narumiya et al., 2000) only inhibited Lp attachment but did not cause a significant defect in bacterial internalization. These results suggest that an alternative pathway for myosin II activation downstream of Rho GTPases could be playing a role in the internalization of Lp filaments. Indeed ROCK independent and MLCK dependent activation of myosin II has been previously reported (Totsukawa et al., 2004; Kassianidou et al., 2017).

Altogether our findings show that filamentous Lp utilizes Rho GTPases for the invasion of LECs. Although hijacking Rho GTPases is a common strategy employed by intracellular bacterial pathogens (Huveneers and Danen, 2009; Hall, 2012), to our knowledge this is the first study that demonstrates that Lp utilizes such a mechanism for the invasion of host epithelial cells.

Interestingly, the activation of Rho GTPases by attachment of filamentous Lp occurs independently of T4SS effectors. Nevertheless, we found that bacterial segments entrapped inside membrane wraps translocated T4SS dependent effectors into the host cells. The primordial membrane wraps formed by the activation of Rho GTPases and the action of Arp2/3 and mDia, may allow enough proximity between the LEC surface and Lp filaments, as well as time for filamentous Lp to inject effectors to manipulate the host cells, while still attached at the cell surface. Indeed, we found that the T4SS effector VipA, an actin nucleating protein (Franco et al., 2012), is needed for the entry of filamentous Lp in LECs. The activity of VipA may complement Arp2/3 and mDia dependent actin nucleation, as membrane wrap morphogenesis and their capacity to elongate are diminished in *dotA* and *vipA* mutants, demonstrating that VipA facilitates a faster uptake of filamentous Lp in LECs. Thus, VipA translocated from the membrane wrap-internalized segments of filamentous Lp could drive the actin polymerization required for membrane wrap elongation and Lp internalization. Of note, only a few other bacterial effectors that can directly nucleate actin have been identified, including *Salmonella typhimurium* effector SipC, which nucleates actin with the same efficiency as Arp2/3 (Chang et al., 2007) and *Chlamydia trachomatis* effector Tarp that can cause formation of actin filaments to mediate bacterial invasion (Clifton et al., 2004).

**Figure 10:**
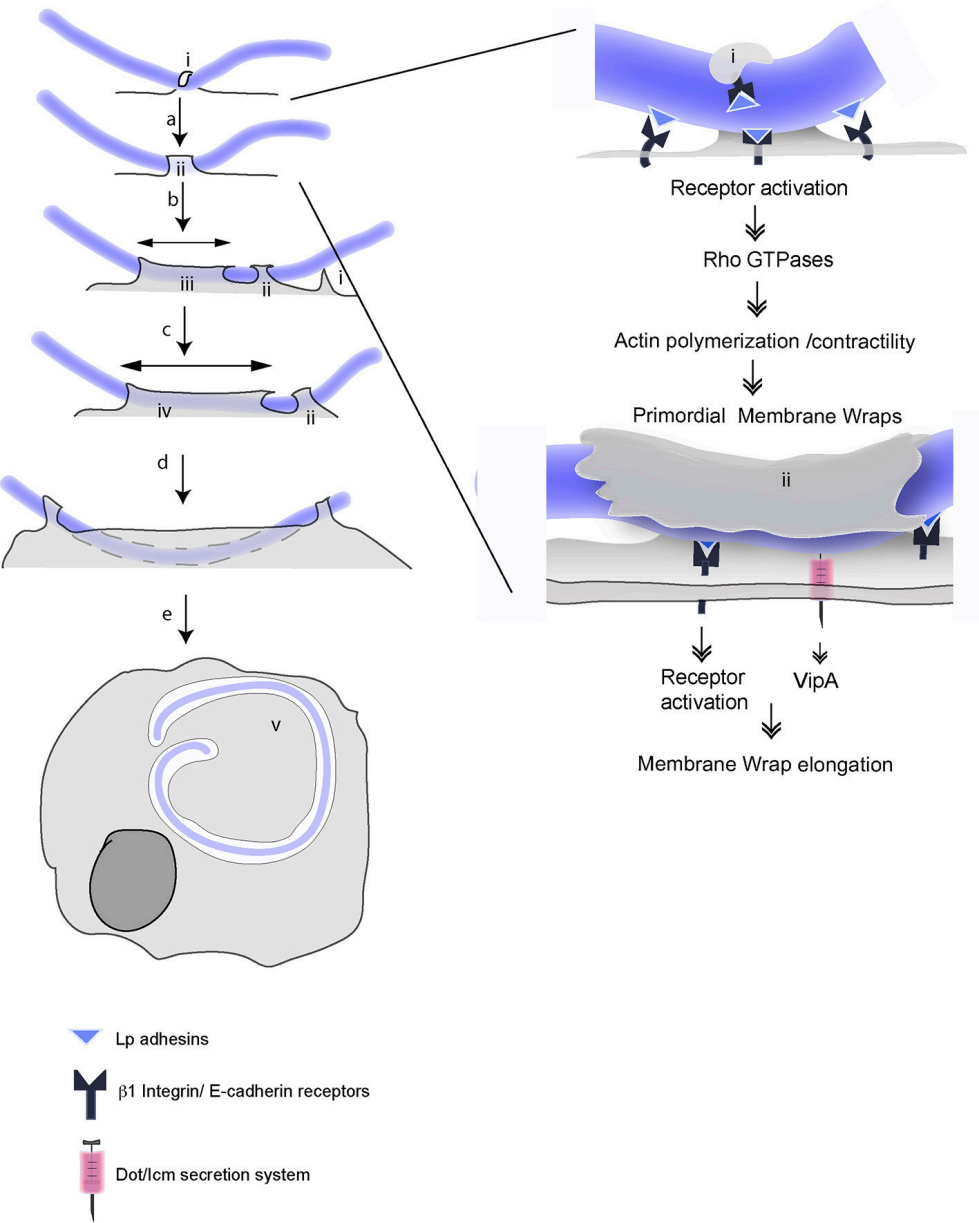
Multistep mechanisms for attachment and internalization of filamentous Lp in LECs. Multistep mechanism for attachment and invasion of LECs by filamentous Lp, according to Prashar et al. (2012) and this study. Unknown Lp adhesins engage host cell β1integrin and E-cadherin receptors at the cell surface and lead to the formation of hooks (i). (a) Downstream signaling initiated in response to receptor activation (SRC and Class I PI3K) switch on small Rho GTPases, which subsequently activate Arp2/3 and mDia dependent actin polymerization and Myosin II mediated actin contraction to form primordial membrane wraps (ii). (See details in the left). The proximity to cell surface allows bacterial segments trapped in the primordial wraps to use T4SS to inject the effector VipA into the host cells (b). Actin polymerization driven by VipA, Arp2/3 and mDia together mediate the elongation of the membrane wrap (iii). This facilitates bacteria-cell contacts, prompting the formation of new membrane wraps that trap adjacent sections of the bacterial filament (c). VipA favors the fusion of adjacent membrane wraps and the internalization of longer segments of filamentous bacteria (d). This process leads to the internalization of the filamentous Lp by LECs where the bacteria reside and multiply in an LCV (v).

Considering our findings altogether, we propose a multi-stage model of LEC invasion by filamentous Lp. The first phase of this process depends exclusively on the bacterial binding to β1 integrin and E-cadherin receptors and their activation to form hooks and primordial membrane wraps that can elongate, but not sufficiently to merge. This initial binding to receptors and the consequent formation of hooks and membrane wraps rapidly entraps the bacteria on the LEC surface (Figure 10). In the lung environment, this would prevent bacterial clearance by the host mucociliary response (Lieberman et al., 1996). The larger surface area presented by filaments could favor the engaging of host cells receptors, and as it has been shown for filamentous *E. coli*, confer an advantage in adhering to surfaces under physiological flow conditions, accelerating bacterial colonization (Möller et al., 2013).

The binding to β1 integrin and E-cadherin receptors, which is required to initiate the first phase of invasion may reduce the likelihood of Lp infecting healthy lung mucosa where these receptors are segregated basolaterally by tight junctions (TJs). Supporting this, our findings show that Lp is unable to break TJs and consequently Lp attachment to polarized epithelial cell monolayers is severely inhibited unless the monolayers are disrupted, exposing the receptors. This is in contrast to professional pathogens including *Burkholderia cenocepacia, P. aeruginosa*, and enteropathogenic *E. coli*, which secrete toxins that can disrupt tight junctions (TJs) (Hanajima-Ozawa et al., 2007; Soong et al., 2008). Importantly, our findings correlate well with clinical data indicating that Legionnaires' disease develops in individuals with underlying lung conditions that compromise tissue integrity (Petecchia et al., 2009; Tam et al., 2011).

In the second phase of the invasion process, Lp filaments, held securely inside primordial membrane wraps are able to utilize their T4SS to translocate VipA and other effectors into the LECs to elongate and modify the membrane wraps into a pre-vacuolar, calnexin positive LCV like compartment. The elongation of a primordial membrane wrap by VipA may favor new close contacts between bacteria and host cell receptors. These contacts will, in turn, allow new primordial membrane wrap to form and elongate to eventually merge by the action of VipA, thus internalizing longer portion of the filamentous bacteria (Figure 10). Is VipA the only T4SS translocated Lp effector mediating the internalization of filamentous Lp? Although for most of the internalization parameters analyzed in this study, *vipA* mutants perform similarly to *dotA* mutants, *dotA* mutants produced more membrane wraps than those generated by *vipA* mutants or wild type Lp. These results suggest that additional T4SS translocated effector(s) may act to limit the number of membrane wraps. It is reasonable to speculate that RavK, a recently described Lp effector that can cleave actin and thereby prevent actin filament formation (Liu et al., 2017) could contribute to membrane wrap morphogenesis. The remodeling of cortical actin required for the loss of membrane rigidity necessary for the invasion of epithelial cells has been described for *Salmonella* (Mason et al., 2007) and *Shigella* (Lhocine et al., 2015). During the course of invasion, the segments of Lp filaments entrapped in membrane wraps lose their association with actin (Prashar et al., 2012). We envision that the coordinated activity of VipA and RavK could be responsible for cytoskeletal remodeling during the internalization of Lp segments. Ceg14 (Guo et al., 2014) and Legk (Michard et al., 2015) are two additional Lp T4SS effectors that have been reported to modulate actin cytoskeleton, however their role in Lp internalization remains to be assessed. Furthermore, several Lp effectors that target host phosphoinositides (PIs) have been described (Qiu and Luo, 2017). For instance, SidF acts as a phosphatase to remove D3 phosphate from PI(3,4,5)P3 to produce PI(4,5)P2 (Hsu et al., 2012), a well characterized regulator of actin polymerization (Hilpelä et al., 2004). While their role has only been studied in the context of LCV formation and maturation, given the critical role of PIs as regulators of actin dynamics, through targeting host PIs, Lp effectors could indirectly modify host actin cytoskeleton needed for membrane wrap elongation.

Bacterial attachment to host cells is the first step in the invasion process. Attachment to β1 integrin and E-cadherin receptors on the LEC surface and the subsequent formation of the primordial membrane wraps anchor filamentous Lp to the cell surface, initiating the invasion process. Both host-mediated responses, namely Cdc42, Rac1, RhoA, and their downstream effectors, and Lp T4SS translocated effectors facilitate the invasion of LECs by filamentous Lp via membrane wraps. While further work is needed to determine the potential contribution of this morphotype to Legionnaires' disease, our findings delineate the molecular details of the mechanism responsible for the invasion of LECs by filamentous Lp.

## Author contributions

AP, CA, CG, and MT conceived and designed the experiments. AP, MO, SL, EB, ZT, FS, and DR performed the experiments and AP, MO, SL, EB, ZT, FS, and DR analyzed the data. AP and MT wrote the manuscript.

### Conflict of interest statement

The authors declare that the research was conducted in the absence of any commercial or financial relationships that could be construed as a potential conflict of interest.
