# Supplementary material for: Small Rho GTPases and the Effector VipA Mediate the Invasion of Epithelial Cells by Filamentous Legionella pneumophila
Source: Front Cell Infect Microbiol. 2018 May 3;8:133. doi: 10.3389/fcimb.2018.00133 (PMC5943596; doi:10.3389/fcimb.2018.00133)
Supplement: Supplementary file 1 [file Data_Sheet_1.pdf]

Supplementary Material

Small Rho GTPases and the effector VipA mediate the invasion of epithelial cells by filamentous *Legionella pneumophila*

Akriti Prashar[1,2] , María Eugenia Ortiz[1], Stefanie Lucarelli[3]¶, Elizabeth Barker[1,2]¶, Zohreh Tabatabeiyazdi[1,2], Feras Shamoun[1], Deepa Raju[1], Costin Antonescu[3], Cyril Guyard[4,5] and Mauricio R. Terebiznik[1,2*]

[1]Department of Biological Sciences, University of Toronto at Scarborough, ON, Canada

[2]Department of Cell and Systems Biology, University of Toronto Toronto, ON, Canada

[3]Department of Chemistry and Biology, Ryerson University, Toronto, ON, Canada

[4]Bioaster, 40 avenue Tony Garnier, Lyon, France

[5]Public Health Ontario, Toronto, ON, Canada

¶ These authors contributed equally to this work

*** Correspondence:**
Corresponding Author
terebiznik@utsc.utoronto.ca

Supplementary Data

FigureS1 Localization of (A) Src-YFP and (B) PH-AKT-GFP. NCI-H292 cells were transfected with the indicated constructs overnight, fixed and imaged. (C) Representative confocal micrographs showing effect of PP2 and Ly294002 on Lp attachment. Cells were pre-treated with PP2 and Ly294002 for 1h followed by infection. Bacteria were allowed to attach for 1h and cells were fixed. All scale bars, 12 μm.

FigureS2 Localization of (A) Cdc42-GFP, (B) Rac1-GFP, (C) RhoA-GFP, (D) PAK-PBD-GFP and (E) rGBD-GFP in NCI-H292 cells. Cells were transfected with the indicated constructs. Following overnight expression of the markers, cells were fixed and imaged.

FigureS3 Disruption of tight junctions in MDCK monolayers following incubation in low calcium media.

Monolayers of MDCK cells were incubated with low calcium media (140mM NaCl, 20mM Hepes, pH7.4, 3mM KCl, 1mM MgCl2, 5mM glucose and 5uM CaCl2) for 1h at 37°C/ 5% CO_2. Cells were moved to a pre-cooled stage and FM4-64 (shown in white) was added to the media. NucBlue live probe (ThermoFisher Scientific) was added to label the nuclei (cyan).

FigureS4 Length of wild-type Lp compared to *dotA* mutants.

Lengths of RFP-Lp or RFP-*dotA* mutants attached to LECs at 6hp.i were measured. Data shown are mean±SEM from 3 independent experiments (n>50 bacteria in each case).

FigureS5 VipA contributes to the internalization of filamentous Lp in LECs

(A) PCR products following the amplification of *vipA* from genomic DNA from wild-type or *vipA* deletion mutants. (B) Western blot showing the expressing of VipA by the indicated bacterial strains. Post-exponential phase bacterial cultures were adjusted to an OD_{600} 2.0. Bacterial pellets were lysed

by addition of loading buffer and resolved by SDS-PAGE followed by immunoblotting with anti-VipA antibodies. (C) Lengths of wild-type Lp and *VipA* mutants attached to LECs. Data shown are mean±SEM from 3 independent experiments (n>50 bacteria in each case). (D) Representative fluorescence micrographs showing the impaired internalization of *dotA* mutants and *VipA* deletion mutants compared to wild-type Lp 12h p.i. Cells were infected as indicated, unbound bacteria washed 1h p.i and cells were fixed 12h p.i. External bacteria were labeled using anti-Lp antibodies followed by Alexa647 conjugated secondary antibodies (blue). Cells were permeabilized, total bacteria were labeled using anti-Lp primary and Alexa 555 conjugated secondary antibodies (red) and F-actin was stained using phalloidin (green). Scale bars, 12 μm. (E) Number of segments undergoing internalization per Lp filament 12h p.i. LECs were infected for 1h, unbound bacteria were washed and infection allowed to proceed for additional 11h. External bacteria were immunolabeled in un-permeabilized cells and the number of Lp segments were enumerated. Data shown are mean±SEM from 3 independent experiments (n>500 cells per experiment). Statistical analysis was performed using one way ANOVA with Tukey's multiple comparison test.

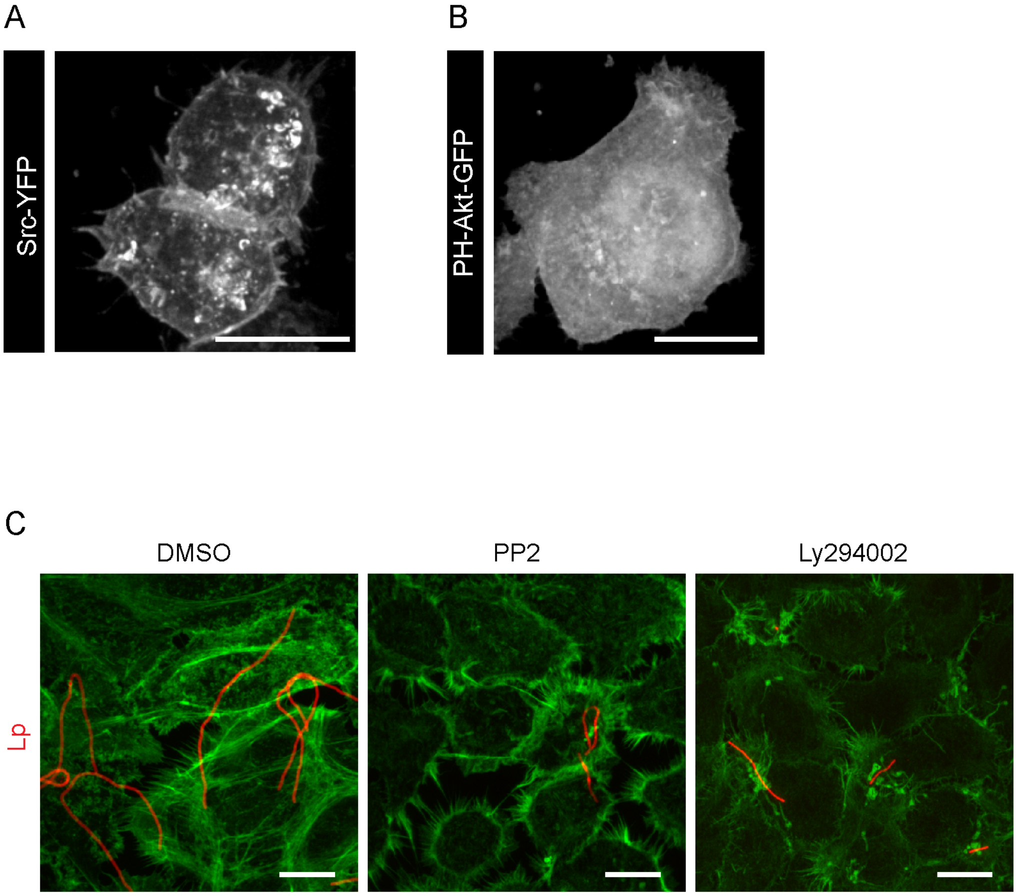

Figure S1

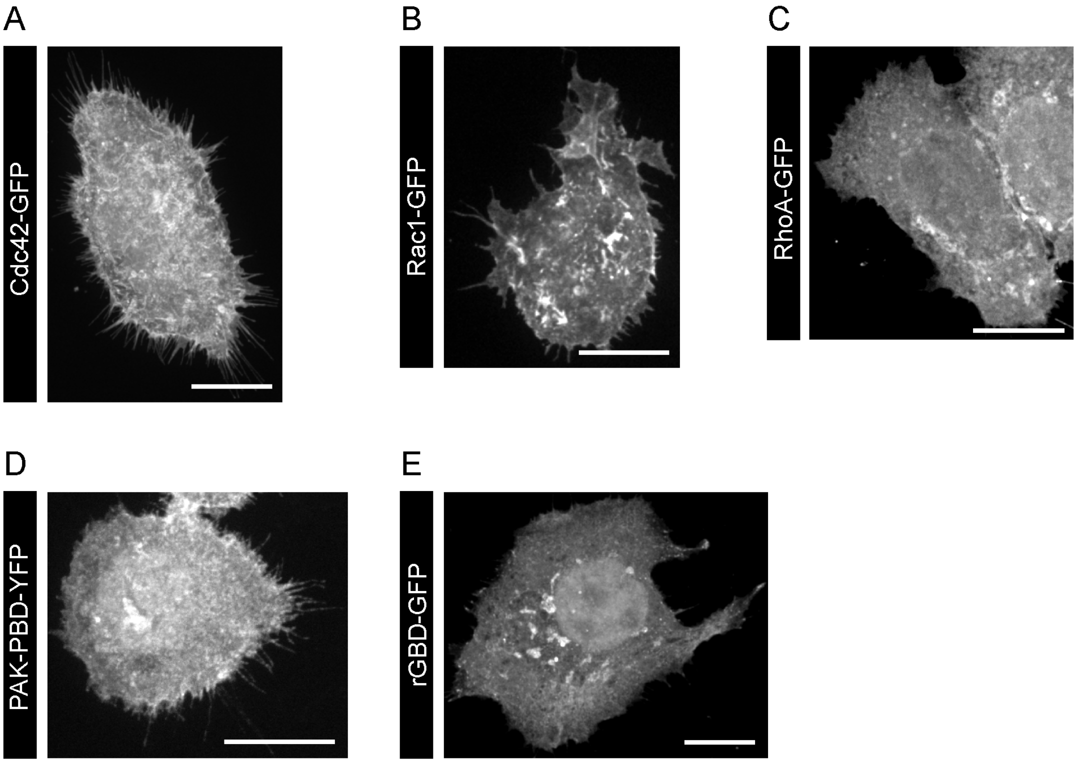

Figure S2

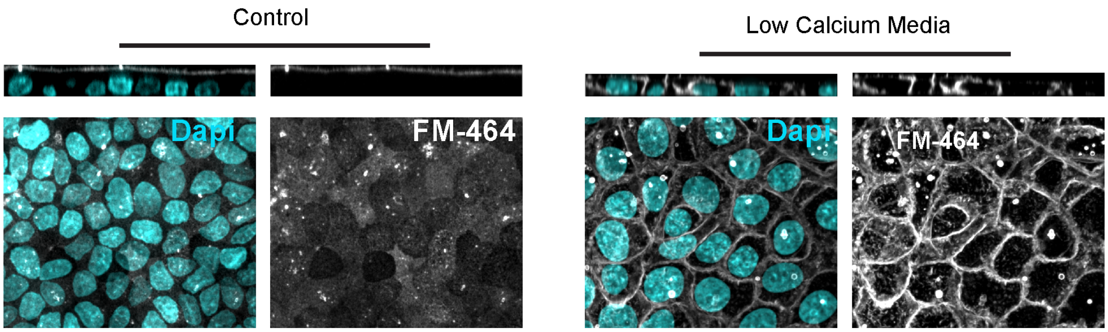

Figure S3

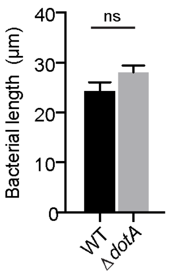

Figure S4

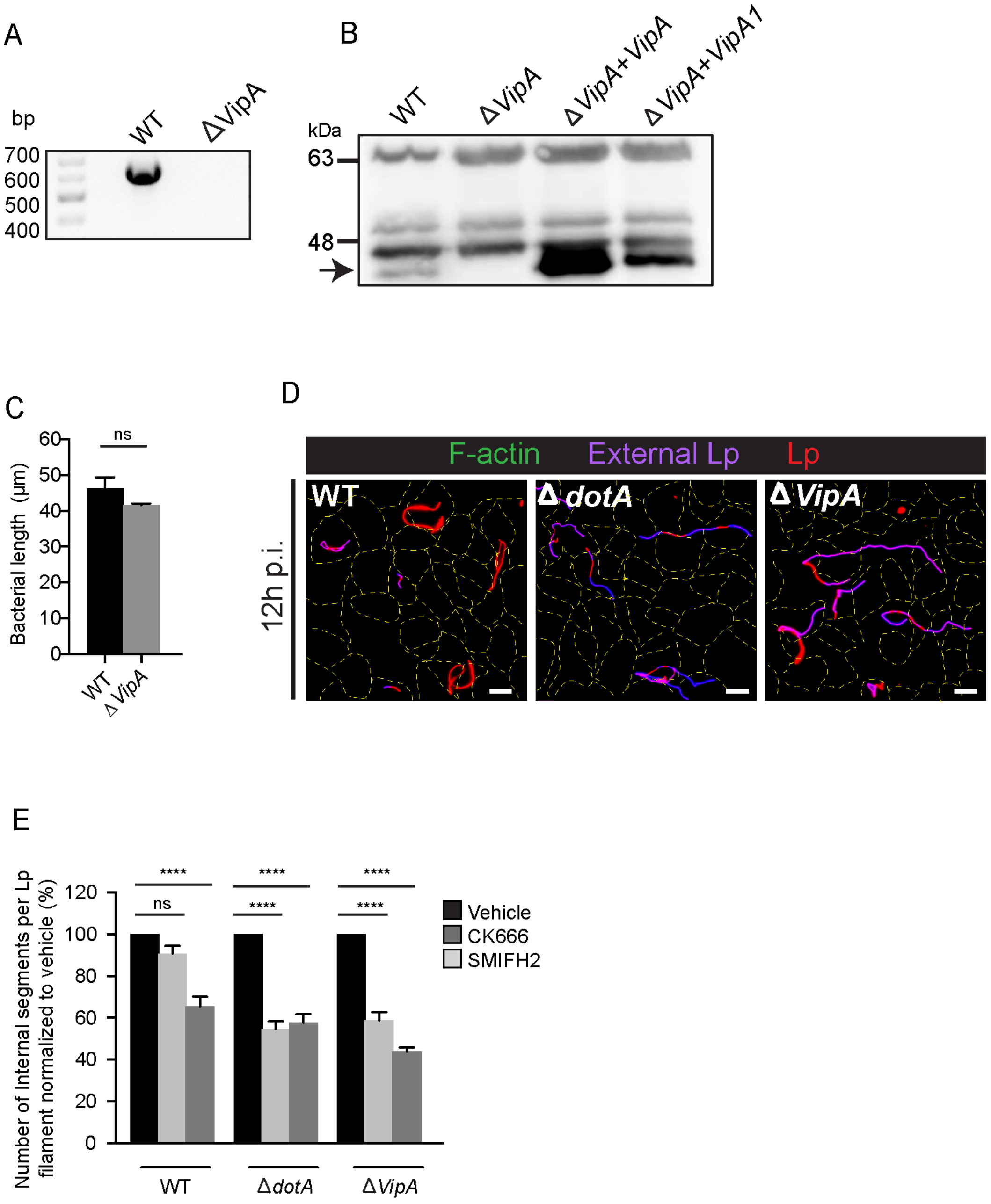
A
bp
WT
ΔVipA
700
600
500
400
B
kDa
WT
ΔVipA
ΔVipA+VipA
ΔVipA+VipA1
63
48
C
ns
Bacterial length (μm)
60
50
40
30
20
10
0
WT
ΔVipA
D
F-actin
External Lp
Lp
WT
Δ dotA
Δ VipA
12h p.i.
E
Number of Internal segments per Lp filament normalized to vehicle (%)
120
100
80
60
40
20
0
ns
****
Vehicle
CK666
SMIFH2
WT
ΔdotA
ΔVipA

Figure S5
